# Disease Spread through Animal Movements: A Static and Temporal Network Analysis of Pig Trade in Germany

**DOI:** 10.1371/journal.pone.0155196

**Published:** 2016-05-06

**Authors:** Hartmut H. K. Lentz, Andreas Koher, Philipp Hövel, Jörn Gethmann, Carola Sauter-Louis, Thomas Selhorst, Franz J. Conraths

**Affiliations:** 1 Institute of Epidemiology, Friedrich-Loeffler-Institute, Südufer 10, 17493 Greifswald, Germany; 2 Institute of Theoretical Physics, Technische Universität Berlin, Hardenbergstraße 36, 10623 Berlin, Germany; 3 Bernstein Center for Computational Neuroscience Berlin, Humboldt-Universität zu Berlin, Philippstraße 13, 10115 Berlin, Germany; 4 Unit Epidemiology, Statistics and Mathematical Modelling, Federal Research Institute for Risk Assessment, Alt-Marienfelde 17-21, 12277 Berlin, Germany; CEFE, FRANCE

## Abstract

**Background:**

Animal trade plays an important role for the spread of infectious diseases in livestock populations. The central question of this work is how infectious diseases can potentially spread via trade in such a livestock population. We address this question by analyzing the underlying network of animal movements. In particular, we consider pig trade in Germany, where trade actors (agricultural premises) form a complex network.

**Methodology:**

The considered pig trade dataset spans several years and is analyzed with respect to its potential to spread infectious diseases. Focusing on measurements of network-topological properties, we avoid the usage of external parameters, since these properties are independent of specific pathogens. They are on the contrary of great importance for understanding any general spreading process on this particular network. We analyze the system using different network models, which include varying amounts of information: (i) static network, (ii) network as a time series of uncorrelated snapshots, (iii) temporal network, where causality is explicitly taken into account.

**Findings:**

We find that a static network view captures many relevant aspects of the trade system, and premises can be classified into two clearly defined risk classes. Moreover, our results allow for an efficient allocation strategy for intervention measures using centrality measures. Data on trade volume do barely alter the results and is therefore of secondary importance. Although a static network description yields useful results, the temporal resolution of data plays an outstanding role for an in-depth understanding of spreading processes. This applies in particular for an accurate calculation of the maximum outbreak size.

## Introduction

Infectious diseases in livestock can spread via various paths. One of the main transmission routes is livestock trade [1–5]. Other transmission routes include direct contact, aerial transmission (e.g. geographical closeness to an index premise) [6–8] and vectors (insect, human, appliances) [9, 10].

Livestock trade is of particular importance, since infectious animals can transmit a disease over long distances between premises. Therefore, massive trade restrictions are implemented in case of an outbreak of a highly contagious disease such as classical swine fever [11]. However, before the first disease case is detected, the disease can spread unrestrictedly via trade. The timespan of this unrestricted trade is called high risk period and can take weeks to months [12, 13]. In addition, trade restrictions are normally not implemented for some endemic diseases such as salmonellosis. Hence, these diseases might freely spread via trade.

The spreading of an infectious disease by trade involves a number of different actors (e.g. farms, slaughterhouses or traders). As these actors form a complex trade system, it is crucial to have an understanding of this trade system. Such systems can be modeled as complex networks.

This analysis focusses on the German pig trade network as a substrate for spreading of infections in pigs. The German pork industry is one of the largest in the world. In the years 2011–2013, Germany was the third largest pork producer after China and the USA [14]. About 4.5 million tons of pork meat are produced in Germany every year. The gain in production value is about 7 billion Euros per year [15]. For the classical swine fever outbreaks in Germany in the 1990s, it has been shown that the most frequent source of infection in secondary outbreaks was the trade with infected pigs [13].

It is the aim of this work to clarify how a disease principally can spread along the German pig trade network. This means estimating the potential transmission ways of a disease between premises connected by direct or indirect trade contacts. In other words, the considered network forms the basis (i.e. the substrate) for disease spread via pig trade in Germany. In reality the spread of infectious disease via trade depends on additional parameters. These parameters can be disease specific (e.g. virulence), farm specific (e.g. biosecurity level) or behavioral (e.g. change in farmers’ behavior due to an ongiong outbreak). Since these specific parameters would bias the principal spreading pathways via trade, we exclude them from our analysis. Hereby, the considered spreading mechanism mimics theoretically possible spreading paths—even if the true transmission probability might be lower for instance due to biosecurity measures.

In order to achieve the aim of revealing potential infection paths in the German pig trade network, the contact patterns between the actors of the system have to be analyzed. In general, contact patterns among hosts forming a contact network are considered as one of the most critical factors contributing to inhomogeneous pathogen transmission, where the assumption of a mass-action process does not hold. During the last decades, veterinary epidemiologists have been focusing on the disease transmission between livestock farms. Premises and animal movements between premises can be translated into nodes and edges of a contact network, respectively.

Techniques adopted from social network analysis (SNA) have been intensively applied by veterinary epidemiologists in order to get a better understanding of the spatio-temporal livestock disease dynamics [1, 16–24] and to identify network actors that are central to the spread of infectious diseases [25, 26].

In order to understand the dynamics of disease spread in complex networks, it is essential to analyze their large scale structure. This is necessary to estimate the size and the incidence rate of a disease outbreak. With this information the consequences of the introduction of a contagious disease can be estimated and control measures can be planned. If nodes and edges differ from one another with respect to their centrality, i.e. to their potential to spread disease, this variability can be used to rank the nodes and edges. Such a ranking allows veterinary authorities to select nodes and edges for the implementation of targeted surveillance and control measures following a central things first rule [27]. In the context of network analysis, the implementation of control measures (such as trade restrictions, vaccination or testing of animals) can be modeled by node removal. This does not necessarily mean that these nodes are not active in the trade network, but rather that they effectively can not transmit a disease to other nodes.

Node rankings can be refined using meta information in form of edge weights. In addition, using the temporal resolution of trade data provides a much more realistic picture of possible outbreak dynamics.

Concerning the German pig trade system as a complex network, there is no systematic characterization of this system in the literature so far. Remarkable exceptions are [1], where a subset of the whole network was analyzed including different production types and [28], where the German pig trade network was analyzed using a data-driven approach. In this work we characterize the static and temporal network of pig trade in Germany as a substrate for spreading processes for the first time.

To achieve the aim of understanding how a disease principally can spread via pig trade in Germany, this article is an attempt to provide a comprehensive picture and characterization of the German pig trade network. In order to give a transparent picture of the network, we hereby avoid the usage of external parameters whenever possible. Therefore, neither explicit disease specific parameters nor specific intervention measures such as trade restrictions are considered.

This paper is structured as follows: first, we briefly describe the data under consideration. In Section *Static Network Analysis* we analyze the pig trade data as a static network, where we characterize the network from a large scale perspective and discuss different strategies for targeted control measures. Section *Weighted Network Analysis* gives a brief overview of the impact of trade volume on the static network results. We consider the temporal resolution of the network data set in Sections *Network as Time Series* and *Temporal Network Analysis*. The network is considered as a time series of uncorrelated snapshots in Section *Network as Time Series*. Finally, we take into account causality for network traversal in Section *Temporal Network Analysis*.

### Data

**Fig 1:**
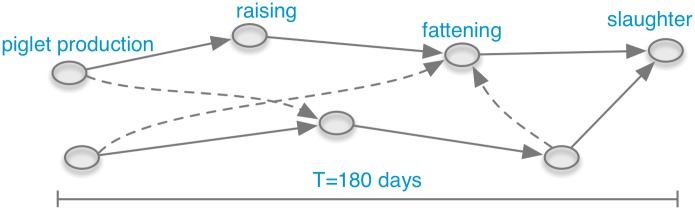
Schematic of pork production chains (solid arrows) in the pig-trade network. Different production chains can be connected by additional cross links (dashed arrows).

In this article we analyze an extract of the HI-Tier database [29]. The database comprises livestock movements of pigs in Germany since 2006. The extract under consideration represents the trade between premises of the pork production chain in Germany in the period between 2011-01-01 and 2014-12-31. Considered data are owner, prepossessor, trade date and trade volume. Thereof a network is generated where trading premises (farms) are *nodes* that are connected by directed *edges* (trade links). In addition, trade volume can be included as further information giving a weight to each edge of the network. The system consists of elementary pork production chains. Fig 1 depicts a schematic illustration of the underlying farming system of the network. The figure shows only the production chain of piglet production, raising, fattening and slaughter. Traders and breeding are not shown.

## Analysis

The resulting network is analyzed in different representations:

1 *Static network*. Direction of trade is taken into account (in principle, the network could also be considered undirected. However, this approach is less meaningful in this context due to the directed nature of the involved production chain). Trade connections are aggregated over time, i.e. the network is static. A trade link is drawn if there is at least one trade action over the observation period. In addition, we analyze the impact of trade volume.
2 *Network as time series*. The system is considered as a time series of directed network snapshots at different time steps. Edge weights are considered to some extent.
3 *Temporal network*. The system is considered as a time series of directed network snapshots at different time steps. In addition, causality is fully considered for network traversal via edge sequences.

In each case the network comprises of 97,980 nodes. The static representation (1.) consists of 315,333 edges. For the temporal cases (2.) and (3.) the data set contains 6,359,697 trade transactions (edges). The observation period is *T* = 1461 *d*, i.e. 1.5 million edges per year.

### Static Network Analysis

In this section, we analyze the trade data as a static network. A static network or *graph* *G* = (*V*,*E*) consists of a set of nodes *V* and a set of edges *E*, where every edge connects a pair of nodes. In the considered network, edges have a direction given by trade. Mathematically, a network can be represented as an adjacency matrix **A** with elements (**A**)_*ij*_ = 1 if there is an edge from node *i* to node *j*, and (**A**)_*ij*_ = 0 otherwise. The total number of neighbors (trade partners) of a node (premise) is called its *degree* or *total degree*. If edge direction is considered, we distinguish between *in-degree* (incoming edges) and *out-degree* (outgoing edges).

#### Large Scale Structure I—Components

In the following, we investigate the component structure of the static network by means of connected components. We will see that the directed nature of trade plays a major role here.

In principle, the outbreak size of any epidemic on a network is limited by the component structure of the network as a worst case scenario. A connected component (or simply *component*) is a subset of nodes *C* ⊆ *V* for which a path exists between any pair of nodes in *C*. A *path* *P*_*i* → *j*_ between two nodes *i* and *j* is an indirect connection between them via arbitrarily many edges without traversing a node twice. Note that for directed networks, *P*_*i* → *j*_ does not necessarily imply *P*_*j* → *i*_. In general there may exist a large number of paths between two nodes. In this article, by path we always mean the *shortest path*, i.e. the *P*_*i* → *j*_ with the smallest number of traversed edges. The average shortest path length in the considered network is 5.5, i.e. on average it takes 5.5 steps to go from a randomly chosen node to another randomly chosen node. The maximum shortest path length is called *diameter* and its value is 18 for the considered network (see Table 1).

**Table 1:** Standard network properties of the static German pig trade network. Diameter and shortest path length are computed for the GSCC.

Property	Value
Number of nodes	97,980
Number of edges	315,333
Edge density	3.2 × 10^−5^
size of GSCC	28%
size of GWCC	99%
diameter	18
av. shortest path length	5.5
path density	0.24

Neglecting the directionality of edges, the network exhibits a giant component, which in directed network is commonly called giant weakly connected component (GWCC). For the considered network, it comprises almost all nodes (see Table 1). This means that virtually all nodes of the network are at least ‘touched’ by trade connections. We find that 99% of all nodes are connected through trade contacts. Nodes not belonging to the GWCC form other components which are only very small islands in the network.

The formation of a giant component is also known as percolation and has been studied in a variety of systems [30–32]. From the statistical point of view, a giant component emerges if the number of edges exceeds a certain threshold [32].

**Fig 2:**
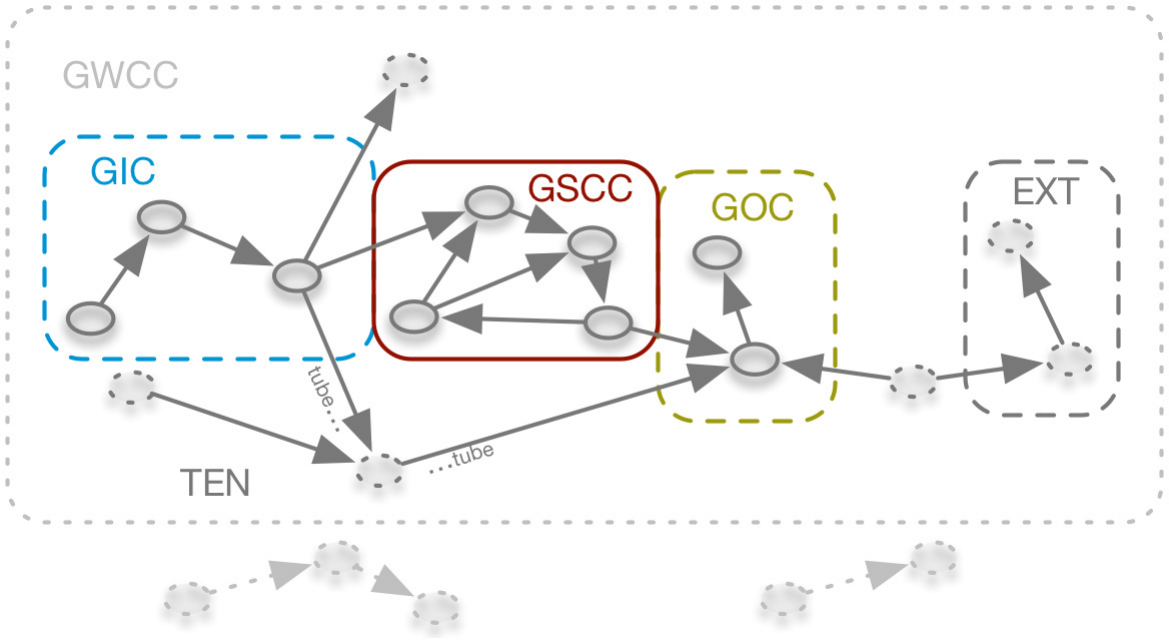
Component structure of directed networks. The giant strongly connected component (GSCC) forms the center of the network (red box). Nodes of the GSCC and the giant in-component (GIC, dashed blue box) have a high spreading potential, whereas all other nodes (GOC—giant out component, dashed yellow box; TEN—tendril; EXT—external nodes, grey dashed box) cannot reach a significant fraction of the network. Box sizes do not reflect the actual sizes of the components. The giant weekly connected component (GWCC) is given by the grey dotted box.

If edge *direction* is taken into account, the network shows a more complex component structure. This is due to the reciprocity that is not guaranteed in directed networks. The general structure of directed networks has been investigated in [31]. It is schematically depicted in Fig 2. In analogy to the GWCC, the *giant strongly connected component* (GSCC) is a subset of nodes for which a *directed* path exists between all pairs of them. For the considered data set, the GSCC contains about 1/4 of all nodes (red box in Fig 2) and forms the backbone of the network in the sense that it ensures the global connectivity of the network. All nodes with access to the GSCC that are not themselves part of it, form the *giant in-component* (GIC, blue frame in Fig 2). In analogy, the *giant out-component* (GOC) contains the nodes that can be reached from the GSCC, but are not part of the GSCC themselves (yellow box in Fig 2).

Besides the mentioned components, a directed network generally contains so-called *tendrils* (TEN, grey dashed nodes in Fig 2). Tendrils are sets of nodes that do not belong to the GSCC, but are reachable from the GIC or that can reach the GOC. A special case of tendrils are *tubes*, which start at the GIC and bypass the GSCC to end in the GOC. Finally, *external node sets* (EXT in Fig 2) are part of the GWCC, but have no access to the GOC.

**Fig 3:**
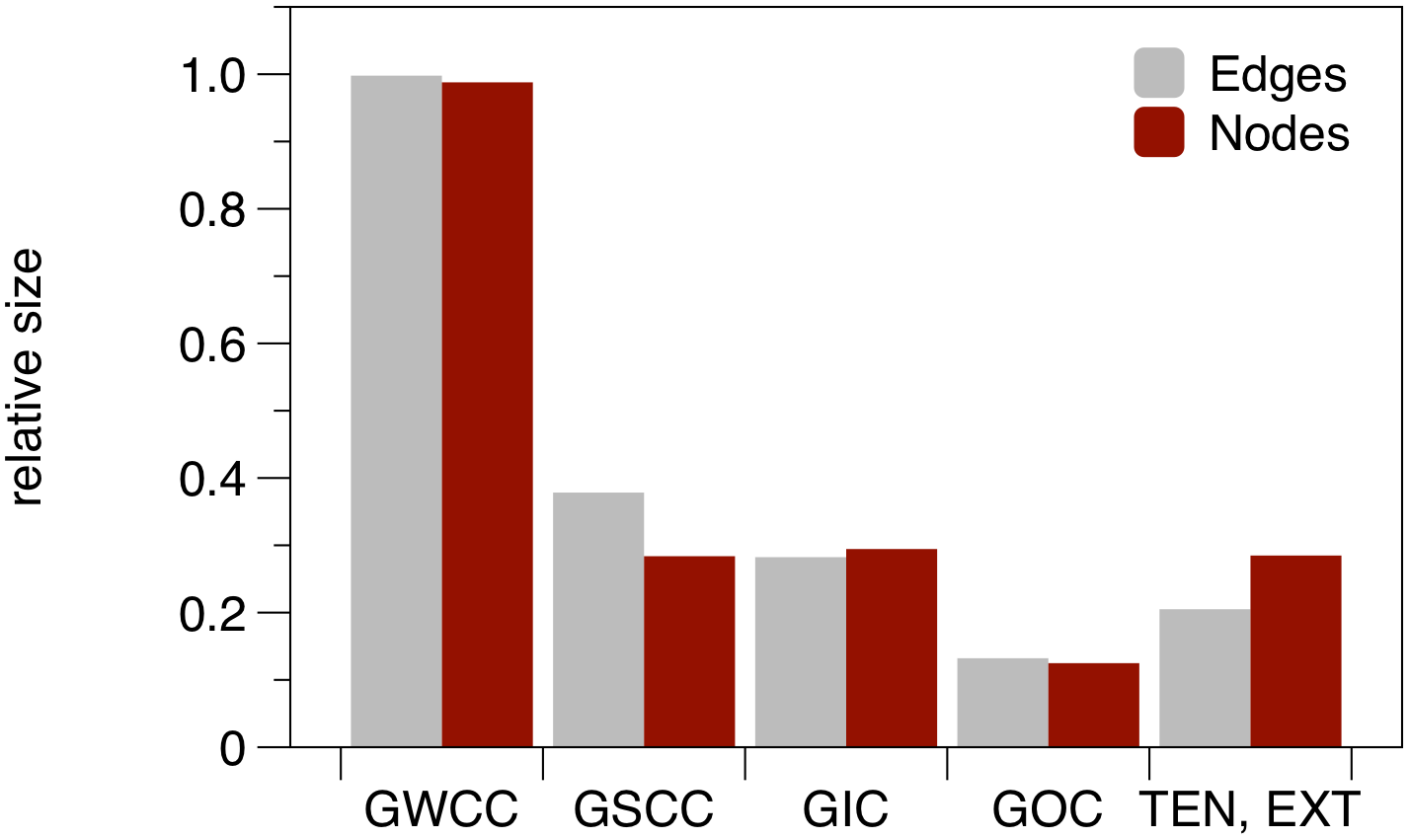
Relative sizes of the large scale components of the pig trade network. Sizes are normalized to the total number of nodes in the network.

Fig 3 shows the distribution of nodes and edges of the different giant structures. About half of the nodes are in the GSCC and the GIC. They form the part of the nodes that can in principle cause larger outbreaks. It is remarkable that the GOC makes up only a small part of the network.

The topology shown in Fig 2 results in a salient property regarding the spreading potential of the nodes in the network. The spreading potential of a node *i* can be quantified using the number of nodes reachable from node *i* by a path of arbitrary length. We call this number the *range* of node *i* [33]. Therefore, the range of a node defines a simple measure for assessing the risk of a pathogen to spread via the network.

**Fig 4:**
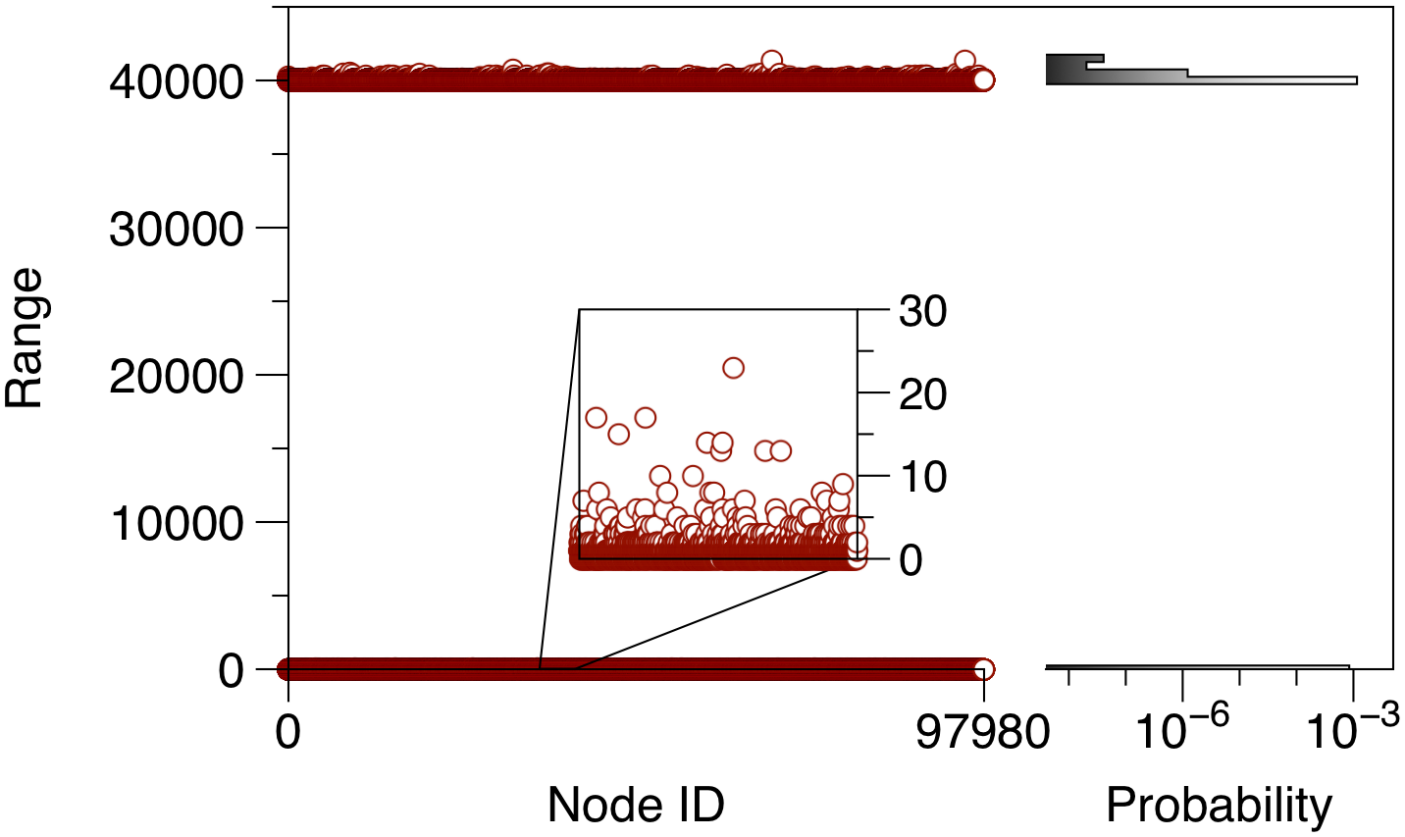
Range for every node in the pig trade network. About 50% of the nodes have a long range of approximately 40,000 nodes, i.e. an infection starting here could reach almost half the network (under the assumption of time-independent links). The other 50% of the network nodes have a short range and cannot cause large outbreaks (maximum short range: 70). The probability distribution of the ranges is shown in the right panel.

Fig 4 depicts the range of every node in the pig trade network. The distribution shows a strong bimodal structure with two node classes: (i) a class with long-range nodes and (ii) a class with short-range nodes. This distribution can be explained with the component structure as described above: The nodes belonging to the GSCC or GIC have a long range. They make up a fraction of 58% (56,656 nodes).

All other nodes (GOC, TEN, EXT) show a considerably shorter range. They represent a much smaller risk for the spread of infectious diseases. On the other hand, the maximum range of all short range nodes is still 70.

In principle, tendrils might form large structures as well. The size of these structures can be estimated by removing the GSCC from the network and computing the ranges of the remaining nodes. The maximum range of the tendril nodes is 48 for the pig-trade network. Nodes of the GOC have a maximum range of 40, whereas EXT nodes can reach up to 70 nodes. Overall, this shows that disease spread via trade in GOC, TEN and EXT cannot cause large outbreaks. Table 2 shows the maximum ranges in the different giant structures.

**Table 2:** Maximum ranges for the large scale structures of the network.

Component	Category	Max. range
GIC	long range	41,369
GSCC	long range	40,040
GOC	short range	40
TEN	short range	48
EXT	short range	70

It should be noted that the observed range behavior (Fig 4) is typical for directed networks. Since (livestock) trade networks are generally directed, a similar behavior can be expected also for other trade networks. Contrary to trade networks, *social* networks, which can be used to model local spreading dynamics, are rather undirected and do not reveal the features shown in Figs 2 and 4.

#### Large Scale Structure II—Mixing Patterns

In the absence of information on the internal contact structure within a population, a widely used assumption is homogenous mixing. This means that every node could be in contact with any other node in the network and there is no selection bias or characteristic interaction pattern. In this section, we will quantify the strength of mixing structures as they are contained in the pig trade data set. These structures can be arbitrary node categories that have to be defined in the first place (e.g. node type, administrative regions, farm size, hygiene status, etc.). In the following we restrict our analysis to categories that are intrinsically linked to the data at hand. These are:

1. Federal state
2. District
3. Municipality
4. Degree (number of trade links).

First, we assign a category *i* to every node in the network, e.g. the federal state in which the node (premise) is located. Then, we compute the number of edges between nodes of the categories (e.g. federal states) *i* and *j*. This can be summarized in a mixing matrix **e** with elements [34]

$$\begin{array}{cc} {\left(e\right)}_{ij}= & \text{fraction}\;\text{of}\;\text{links}\;\text{between}\\ & \text{categories}\;i\;\text{and}\;j, \end{array}$$ (1)

where the indices *i* and *j* represent different node categories.

The propensity of a network to prefer links between nodes of the same category can be quantified using the assortativity coefficient *φ*. We first focus on the assortativity coefficient for enumerative node categories such as Federal state, District or Municipality. The degree being a scalar node property is discussed below. The *enumerative assortativity coefficient* is defined as follows [34]:

$$\varphi =\frac{\text{Tr}\;e-∥e^{2}∥}{1-∥e^{2}∥},$$ (2)

where Tr **e** = ∑_*i* = *j*_ *e*_*ij*_ is the trace of matrix **e** and ‖**⋅**‖ is the sum over all matrix elements. Formally, *φ* is a correlation coefficient. If a network exhibits a positive assortativity coefficient *φ* > 0, it is called an assortative network (with respect to that category); for *φ* < 0 the network is called disassortative. Networks where *φ* = 0 are called uncorrelated.

In a perfectly assortative network (*φ* = 1) all nodes are connected only to nodes of the same category, e.g. links are only formed between nodes of the same federal state or the same degree. This would correspond to a mixing matrix **e** with finite elements only along the main diagonal, all other elements being zero. On the other hand, the (enumerative) assortativity coefficient for a perfectly disassortative network is in general greater than −1 and the exact value depends on the number of considered categories [34].

Table 3 shows the assortativity coefficients for the four node categories mentioned above. The membership to a federal state represents a large scale classification for each node and the corresponding assortativity coefficient (*φ* = 0.81) is relatively high. Premises have thus a preference to trade within the same federal state. As a consequence, imposing trade restrictions along the borders of federal states results in a rather small modification of the original network in case of an outbreak [35]. Even though trade restrictions along the borders of federal states would disconnect the network, most trade connections are not affected, since the majority of trade connections connects node pairs in the same federal states.

**Table 3:** Assortativity coefficients *φ* of the static German pig trade network. The network is assortative with respect to federal state, district and municipality and disassortative with respect to node degree. *σ* is a statistical error estimate. Assortativity coefficients for node categories are computed using Eq (2), for the degree Eq (3) has been used.

Category	*φ*	*σ*
Federal state	0.81	7 × 10^−4^
District	0.43	2 × 10^−4^
Municipality	0.14	6 × 10^−5^
Degree	-0.13	1 × 10^−3^

**Fig 5:**
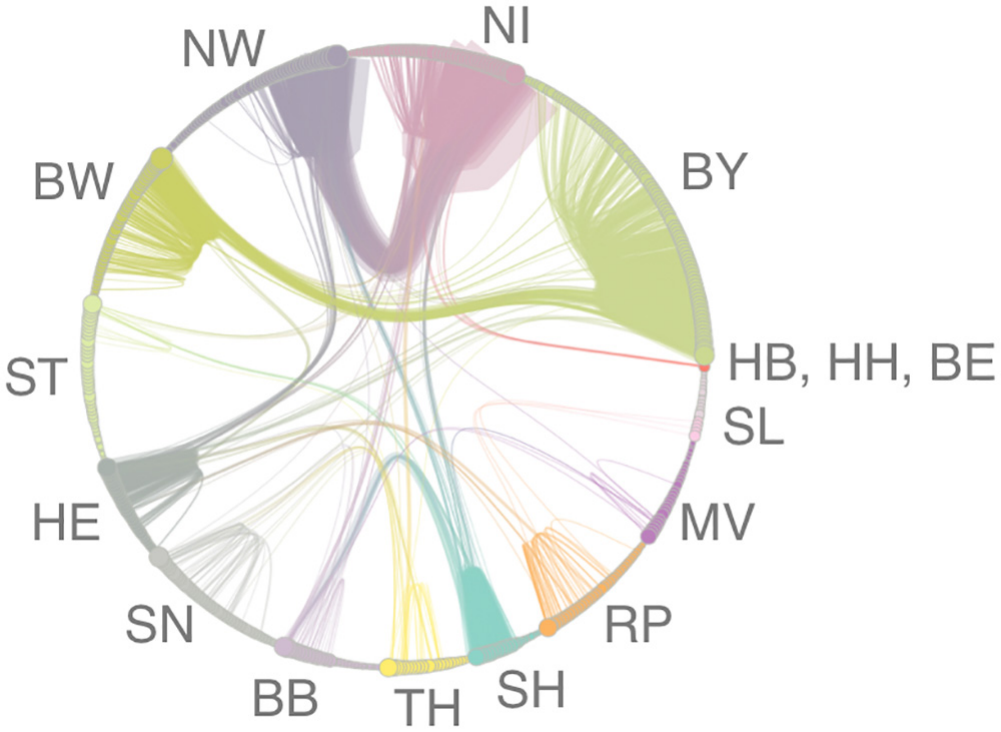
Trade between districts in Germany. Node sizes correspond to the degree. Edges are bundled with respect to the federal states. Trade is dominated by links between districts in NW and NI and BY and BW, respectively. Self-loops (intra-state trade) are not shown.

Fig 5 shows the contact structure between the federal states in Germany. Nodes are districts that are color-coded according to their federal state membership. Intra-district trade links (self loops) are not shown. Edge widths correspond to the number of trade links between two districts. Node sizes correspond to the node degree. For an improved visualization, edges are bundled for each federal state using the algorithm proposed in [36].

We observe again that the majority of trade takes place within the federal states (intra-district trade links not shown in Fig 5). Inter-state links are mainly formed between North Rhine-Westphalia (NW) and Lower Saxony (NI) as well as Bavaria (BY) and Baden-Wuerttemberg (BW). In fact, the trade between NW and NI alone accounts for 36% of all inter-state trade connections. The federal states NI, NW, BW und BY account for 47% of all inter-state edges. Considering the districts of all federal states, 20.5% of the districts contain 80.4% of the inter-state trade links.

Contrary to federal states, there is less tendency that premises trade within their district or municipality (see Table 3). This may originate from the fact that not all types of premises needed for pork production are present in all districts or municipalities.

We now focus on the assortativity with respect to the node degree. It is insightful to investigate whether or not nodes show a tendency to connect with nodes of similar degree. If for example nodes of high in-degree preferably trade with nodes of high out-degree (disassortative network), disease spread would be (at least locally) facilitated. It has been shown that targeted node removal has a stronger effect in disassortative networks than in uncorrelated or assortative ones [37].

In contrast to the enumerative categories discussed above, the degree is a scalar assigned to every node in the network. This implies that not only node pairs with exactly the same degree contribute to the degree assortativity coefficient, but also node pairs of similar degree. Degree assortativity is therefore not analyzed using Eq (2), but is rather measured in a different way. Following [34], the *degree assortativity coefficient* is given by a Pearson correlation coefficient

$$\varphi =\frac{\sum_{xy}\;xy\left(e_{xy}-a_{x}b_{y}\right)}{\sigma_{a}\sigma_{b}}$$ (3)

where *e*_*xy*_ are the entries of a mixing matrix containing the fraction of edges connecting nodes of degree *x* and *y*, *a*_*x*_ = ∑_*y*_ *e*_*xy*_ and *b*_*y*_ = ∑_*x*_ *e*_*xy*_. *σ*_*a*_ and *σ*_*b*_ are the standard deviations of *a* and *b*, respectively.

The pig trade network is disassortative with respect to the degree. Table 3 shows the assortativity coefficient for the total degree, which is smaller than zero. Therefore, there is a tendency that nodes of different degree are connected. This behavior is typical for technological and biological networks [37]. In our context, the disassortative degree mixing can be explained firstly by the fact that slaughterhouses receive animals from a large number of different farms (large degree), including many small ones (small degree) (see Fig 1). A similar pattern is formed for piglet production, where few piglet producers provide piglets for a large number of different farms. It is well known that the number of piglet-producers and slaughterhouses is small compared to the rest of the system [38, 39].

Applying control measures in slaughterhouses might be considered effective, but has a minor effect in protecting the rest of the network. On the other hand, applying control measures (e.g. vaccination) at the level of piglet producers is a clearly effective, but rather obvious and trivial strategy. In order to get an estimate for the center of the production chain, we compute the degree assortativity coefficient for the subnetwork where all nodes with vanishing in degree or out degree are removed. Although this procedure is not exact, it should remove most slaughterhouses and piglet producers from the network. The assortativity coefficient for this subnetwork is −0.16 and thus this part of the network is still disassortative with respect to the degree.

Besides the total degree, we also compute *φ* for all combinations of in-degree and out-degree. The values are similar to the total degree case and are shown in Section A in S1 File.

The standard deviation *σ* of the mixing coefficient can be estimated statistically [34]. For the considered network all statistical errors are orders of magnitude smaller than the assortativity coefficients (see Table 3). Thus, the observed results are highly statistically significant.

In conclusion, it follows from the analysis of mixing patterns that federal states provide an intrinsic partition of the network, even if this partition is large-scale. In this context, also several federal states could be combined into even larger clusters. Concerning the local mixing structure of the network, we expect that efficient targeted control measures are possible, since high-degree nodes tend to be connected to many low-degree nodes. Hence, this analysis can contribute to define regions according to the OIE terrestrial animal health code [11].

#### Small Scale Structure—Centrality and Intervention Allocation

In order to implement efficient measures of disease control and surveillance, the infection risk of every node has to be assessed. For this purpose, so-called centrality measures have been defined. The range defined above is one such centrality measure. We have already shown in Table 2 that the range allows us to identify two risk classes for the nodes in the network.

**Fig 6:**
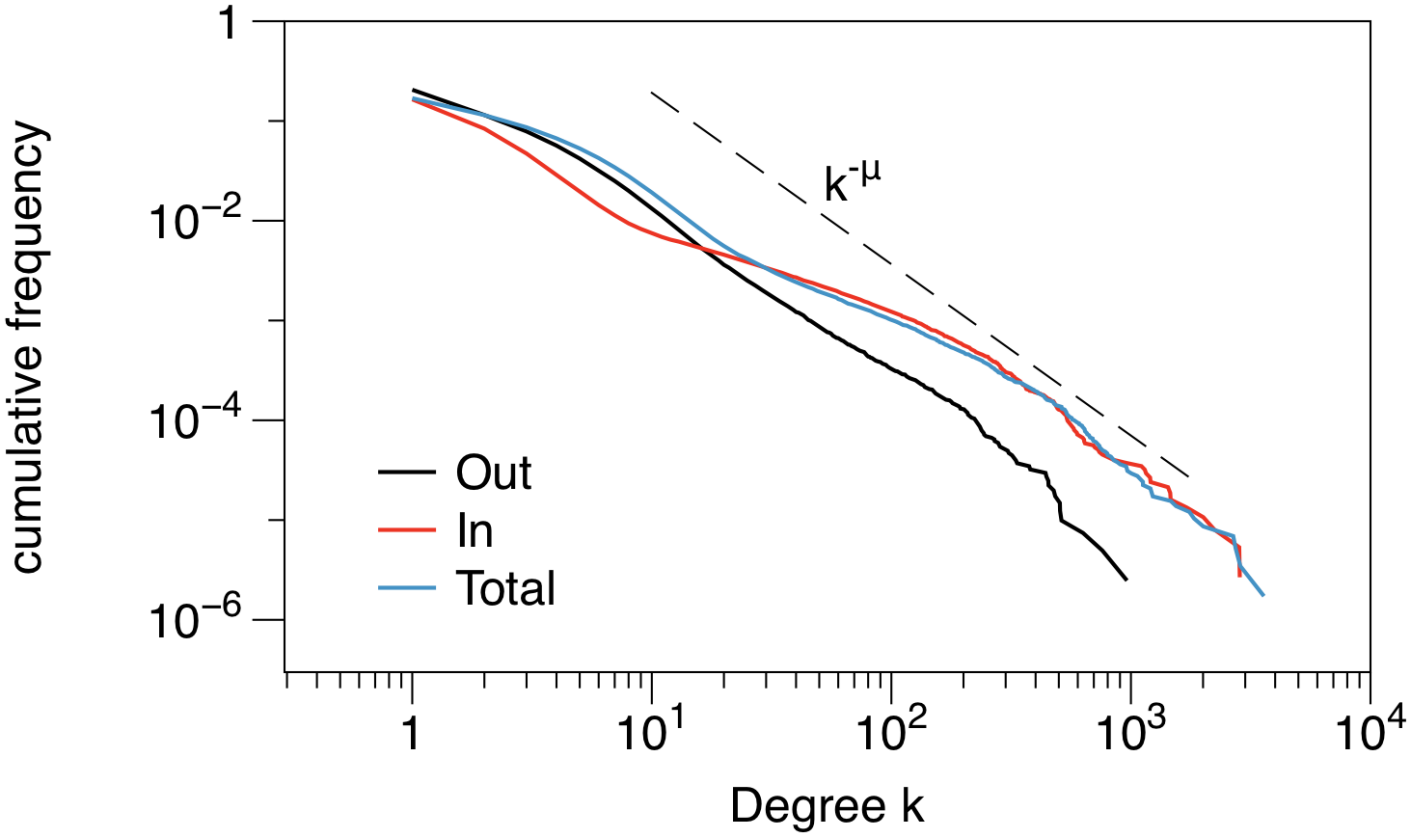
Degree distribution of the livestock trade network. The out-degree distribution can be approximated by a power law of the form *p*_*k*_ ∼ *k*^−*μ*^ with *μ* ≈ 2.7 (estimated using a maximum likelihood approach [49]). The figure shows the cumulative distribution to minimize fluctuations.

The simplest centrality measure is the *degree* of a node, which can be easily obtained by counting the number of neighbors. In the case of the pig trade network, we distinguish between in-degree and out-degree; the total number of links a node is connected to is the total degree *k*. Fig 6 shows the degree distributions of the network. The figure shows the cumulative distribution on log-scale. The distributions are heavy tailed and the out-degree distribution can be approximated by a power-law, i.e. the distribution has the asymptotic form *p*_*k*_ ∼ *k*^−*μ*^ with some constant *μ*. On the other hand the in-degree distribution exhibits a bimodal structure reflecting the existence of large slaughter houses. It has been shown that the degree distribution has a significant impact on disease dynamics [40–48].

In order to investigate different disease control strategies (such as trade restrictions or culling) for the network, the following centrality measures have been computed:

**degree centrality.** *C*_*D*_—Number of neighbors of a node. Normalized to the number of nodes in the network.
**betweenness centrality.** *C*_*B*_—Frequency that a node lies on a shortest path between other nodes.
**closeness centrality.** *C*_*C*_—Reciprocal average shortest path length between a node and all other nodes.

We also investigate other measures such as eigenvector centrality, pagerank und Katz centrality. These measures, however, turn out to be less suited for disease control (see Section B in S1 File). An overview of the role of different centrality measures for disease control is provided in [17] and [16].

Knowledge of the centrality distribution over the network can be used to implement targeted intervention measures. For this purpose nodes are first of all ranked according to their centrality. Then the impact of the removal of nodes with the highest rank on the functionality of the network can be measured. After each node removal, centrality has to be computed again. In our context, removing nodes does not necessarily mean that they are not active in the trade network, but rather that they effectively can not transmit a disease to other nodes. This can be achieved by culling, isolation of animals, increased hygiene measures or vaccination. The functionality of a network can be defined by the size of its GSCC, since the key feature of every network—namely to ensure the interconnectedness between the nodes—is manifested here. If the size of the GSCC is reduced, the network disintegrates into smaller components and every disease outbreak is restricted to small ‘islands’.

The impact of different node removal strategies on networks has been analyzed in [42, 50]. The degree of a node has been shown to be a good indicator for its importance. Furthermore, the degree is relatively easy to measure even if network data knowledge is limited. In addition to the degree we study the suitability of the other centrality measures mentioned above for a risk ranking.

**Fig 7:**
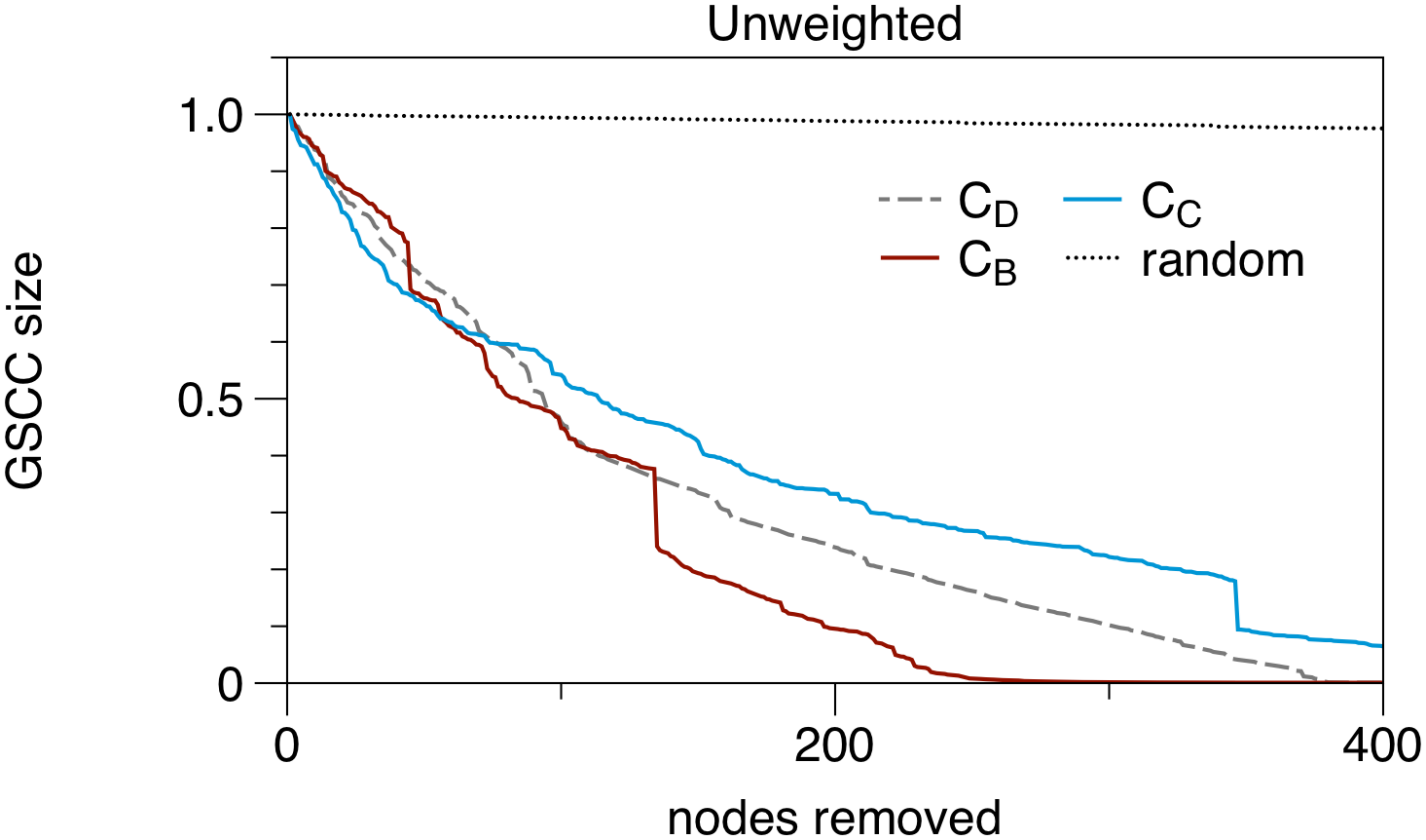
Impact of centrality based node removal, when up to 1% of the nodes are removed. *C*_*D*_-Degree centrality, *C*_*B*_-Betweenness, *C*_*C*_-Closeness. Size of giant strongly connected component is normalized to unity.

Fig 7 shows the size of the GSCC depending on the number of removed nodes; nodes are removed according to their centrality rank in decreasing order.

It is remarkable that the removal of randomly chosen nodes barely has a measurable impact on the functionality of the network (dotted line in Fig 7). This phenomenon has also been observed for other systems [42] and is related to the degree distribution of the network. In the case of random removal, about 30,000 (of all 97,980) nodes must be removed in order to halve the size of the GSCC. For comparison: in case of targeted removal of central nodes it suffices to remove only about 100 nodes to achieve the same effect.

It is apparent from Fig 7 that the optimal strategy depends on the number of nodes to be removed. In practice this number depends on the specifications of disease control. Depending on the number of removed nodes, we define the optimal strategy as the one with the smallest value of the GSCC size at this point (Fig 7). If about 50 nodes are to be removed from the network, nodes of high closeness or degree should be chosen. In case of removing 100 or more nodes, nodes of high closeness are less efficient. In this case, nodes of high degree and above all nodes with high betweenness should be removed. Overall, betweenness centrality shows the best performance for disease control.

The large-scale structure of the network is also apparent in the distribution of centrality measures. Nodes in the GIC tend to show a high out-degree and low in-degree, whereas the opposite holds for the GOC. One could expect that many high in-degree nodes (slaughter houses) are located in the GOC. Interestingly, nodes with the largest in-degrees are located in the GSCC. These results are provided in Section C in S1 File.

We conclude:

1. any centrality based intervention performs significantly better than random intervention.
2. removal of high closeness or degree nodes is efficient for removal of up to 50 nodes.
3. removal of high betweenness nodes is efficient for removal of more than 100 nodes.

### Weighted Network Analysis

In principle, the number of traded animals plays an important role for the spread of animal diseases, since in reality hardly ever all animals in the outgoing farm are infectious. Depending on the trade volume, this could have a strong impact on the epidemic conductivity. Here we distinguish between the *infection probability* and the *infectiousness* of each edge in the network.

For highly contagious diseases (such as classical swine fever, Aujeszky’s disease, foot and mouth disease) a trade contact is even infectious if a single infected animal transported. Thus, the relevant measure here is the *probability* that for a trade contact with another node at least one animal is infected. This probability depends on the trade volume and the prevalence in the originating farm (see Section D in S1 File). Given the relatively high trade volume in the network analyzed here, the mean transmission probability of a trade connection is close to 1 (see Section D in S1 File).

For lowly contagious diseases (such as Tuberculosis) the *infectiousness* of a trade contact plays a central role. Contrary to the infection probability discussed above the infectiousness of a link is closely related to the number of transported animals. Thus, the link weight plays a central role here.

Although the weighted network is topologically similar to the unweighted network, there is evidence that the shortest path structure of the network is different for the weighted case. In fact, we find an average shortest path length of 9.7 and a diameter of 30 for the weighted network. Compared to the static network, both the average shortest path length and the diameter are twice as high (see Table 1). This implies that weighted shortest paths differ from purely topologically shortest paths.

Nevertheless, this circumstance does not seem to alter our findings for the unweighted network. We analyze the weighted network with respect to targeted node removal in see Section D in S1 File. The results found are qualitatively similar to the results of the previous section. In brief, in the context of intervention measures, removing nodes with high weighted degree (i.e. trade volume) turns out to be an appropriate strategy. Additionally, weighted closeness and weighted betweenness perform well as in the unweighted case. Their performance is, however, not superior to the unweighted case.

### Network as Time Series

In the previous sections, time in the data was aggregated over the whole observation period of four years. This section is devoted to the temporal development of the network. Since the trade system changes over time, we first consider the network as a time series of network snapshots. The temporal resolution of the data set is Δ*t* = 1*d*. The time series of the network is given by a sequence of *T* adjacency matrices

$$A=A_{1},A_{2},\ldots ,A_{T},$$ (4)

where *T* is the observation period (here: *T* = 1461 *d*) and each matrix **A**_*t*_ is the adjacency matrix of the network at time *t* (snapshot), i.e. it contains the very edges being active at that time.

The static network analyzed above is given by the aggregation over Eq (4). Thus the adjacency matrix of the static network is

$$A=\sum_{t=0}^{T}A_{t}.$$ (5)

**Fig 8:**
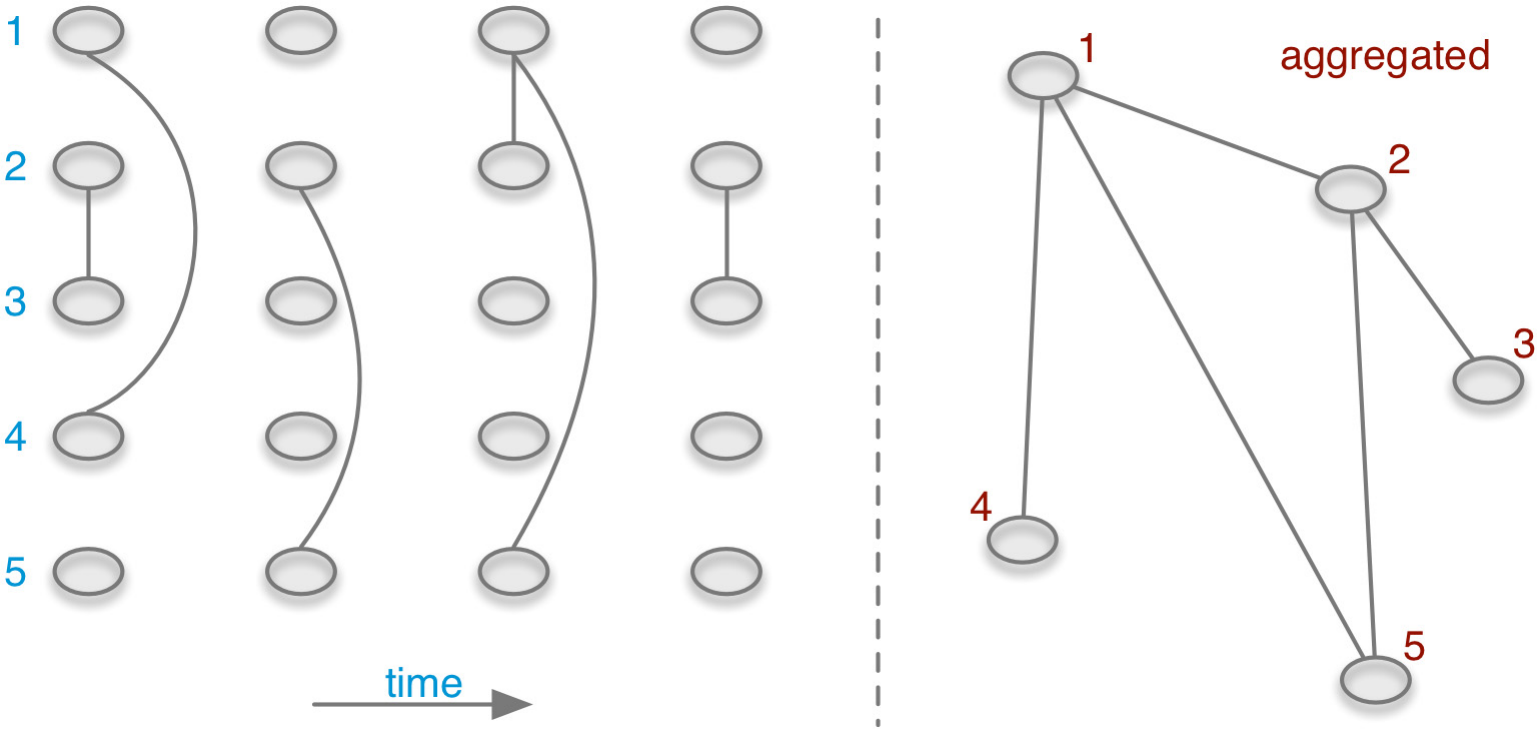
A temporal network (left) and its static counterpart (right).

This directly applies for the aggregation of trade volumes: for the case of the unweighted network, where volume is not taken into account, the matrices can be treated as Boolean. This means in effect that every non zero element is set to unity. Fig 8 shows the aggregation of an exemplary undirected network.

This raises the question how many snapshots **A**_*t*_ have to be aggregated (size of the aggregation window) in order to recover the properties of the static network as discussed above. The minimum aggregation window has been analyzed for a similar data set (supporting information in [28]). It is roughly one year.

**Fig 9:**
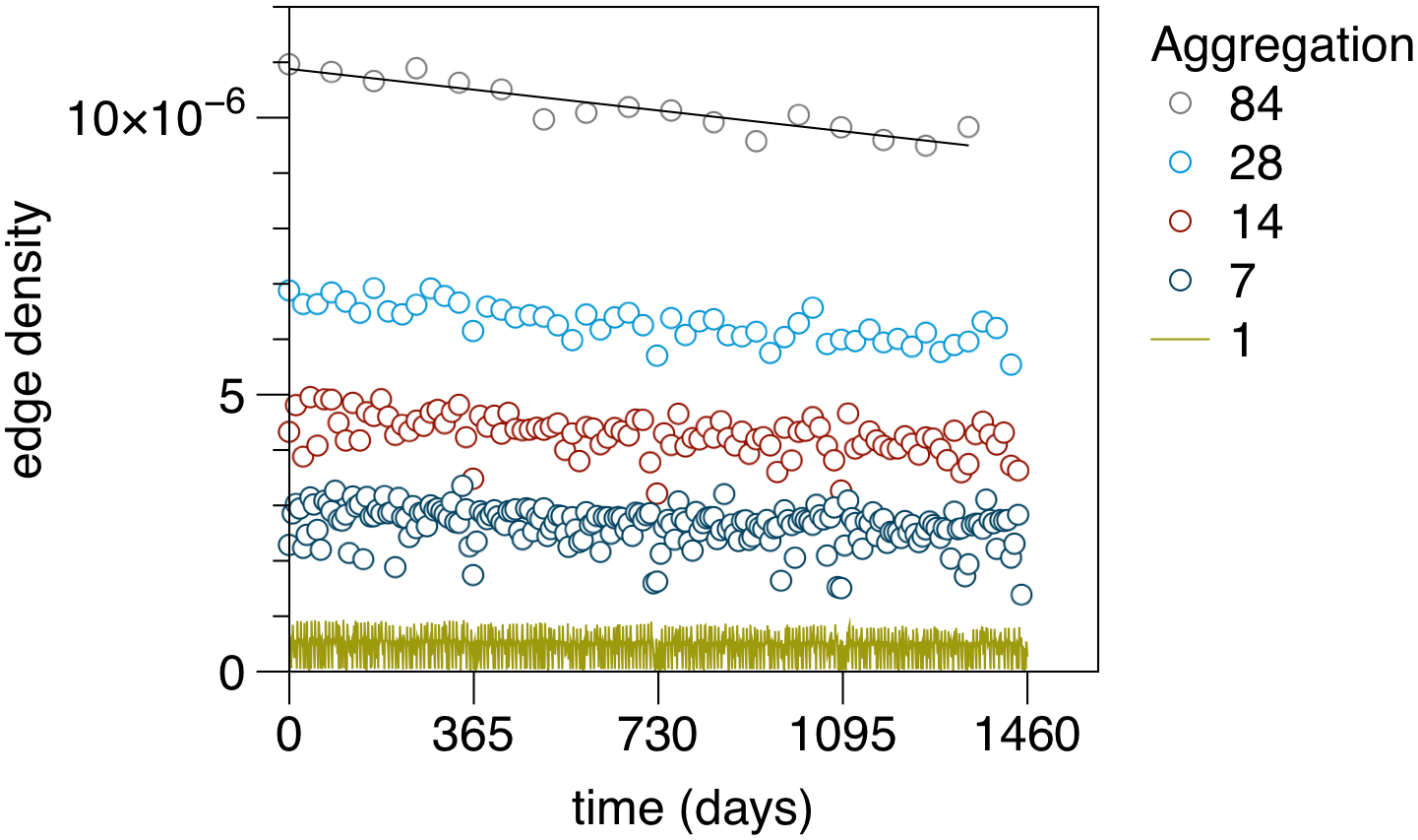
Development of the edge density. There is a clear trend to edge reduction over time. The edge density of the static network is 3 × 10^−5^.

In order to reveal general trends in the temporal evolution of the system, snapshots of the network can be compared at different times. The temporal evolution of the number of edges is shown in Fig 9 (the figure shows the edge density, i.e. the number of edges normalized by the number of theoretical possible edges). To reduce noise in the data, snapshots have been partially aggregated. The following partial aggregations have been considered: 1 d (yellow), 7 d (weekly, dark blue), 14 d (red), 28 d (monthly, light blue), 84 d (quarterly, grey). For an aggregation window of 84 days we find a linear slope of 10^−9^ *d*^−1^; this corresponds to a decrease of about 3,600 edges per year. The number of active nodes shows a similar trend (Section E in S1 File). A decrease in the number of active nodes implies that gradually less premises will play a role for disease spread.

Concerning the difference between the static network and the time series, Fig 9 shows that the edge density of each snapshot, represented by a matrix **A**_*t*_, is on average less than 10^−6^. On the other hand, the static network has an edge density of 3 × 10^−5^ (Table 1). Hence the edge density of the aggregated network is about an order of magnitude higher, i.e. about 10% of the edges are active every day.

**Fig 10:**
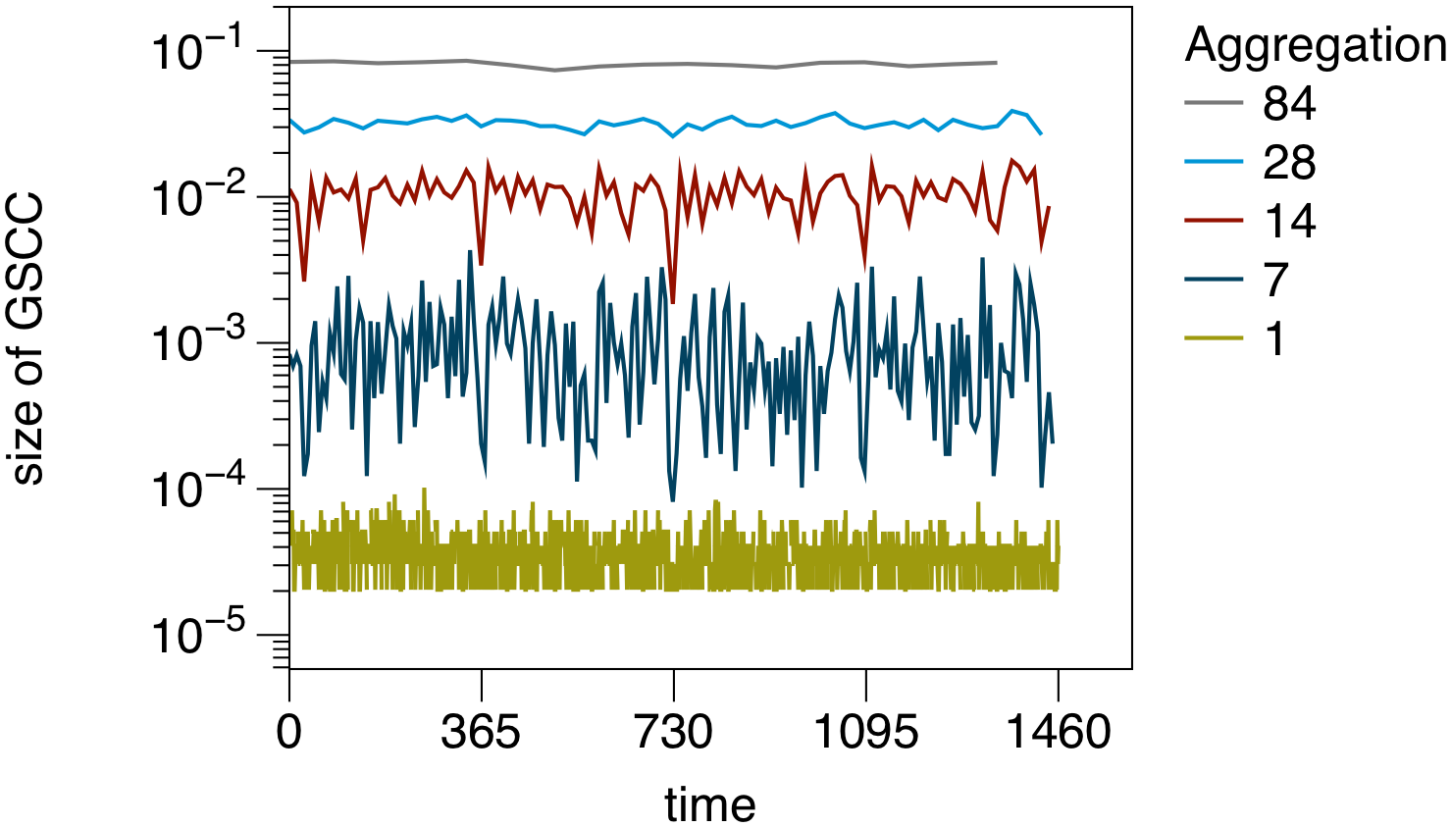
Relative size of the GSCC for different partial aggregation windows. The sizes show stronger seasonal fluctuations on small time scales (red), but remain rather constant over large time scales (grey). Size is normalized by the number of nodes in *G*.

It should be noted that the size of the GSCC is almost unaffected by the trend observed in Fig 9. This is shown in Fig 10. The GSCC shows seasonal fluctuations for intermediate aggregation windows (see also [46]). The relatively high stability of the GSCC over time reflects the fact that the network maintains its functionality even though the number of links is reduced over time.

**Fig 11:**
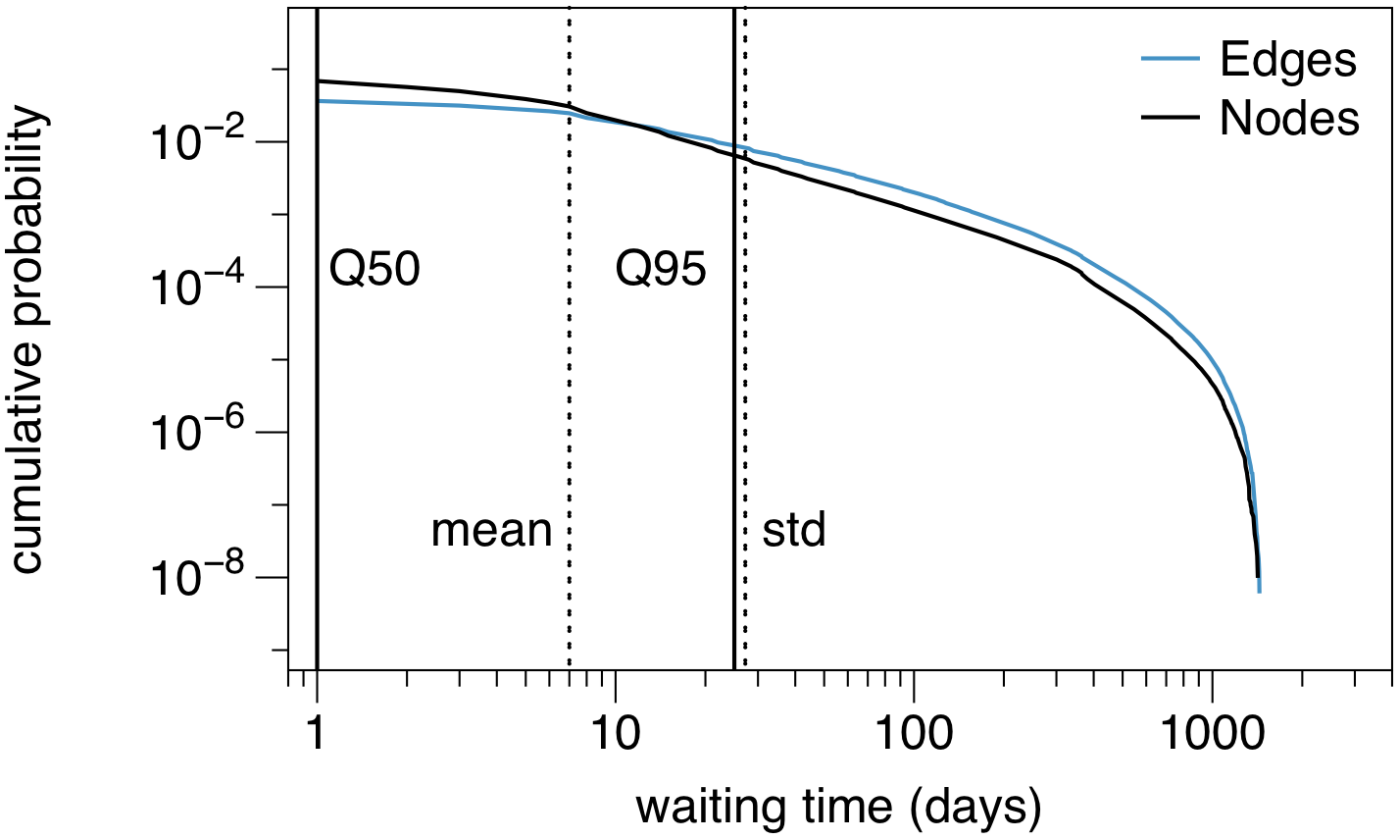
Distribution of node and edge waiting times. The empirical waiting times cover values over three orders of magnitude. Dashed lines show mean and standard deviation of the node waiting times, respectively. Solid lines show median and 95% quantile of the node waiting time distribution.

It is important to note that the waiting times in the network are strongly heterogeneously distributed. The waiting times of a node (or edge) are times where the node (or edge) is not active. Fig 11 shows the distribution of the waiting times of nodes and edges. The figure illustrates that the measured inactivity time spread over several orders of magnitude. Given the shape of the distribution no appropriate mean value can be given for the waiting times. This is also an indication that an interpretation of the trade links as *rates* between nodes (e.g. the flux of animals between node *i* and *j* is *m* animals per day) is not appropriate for this system. A similar behavior has also been found for other system and is referred to as *bursty behavior* [28, 51–53]. It should be mentioned that for the pig trade network considered here, typical waiting times might be in the system, if farm types were resolved in the data. However, this is not the case for the considered data set and thus the inactivity time reflect a global behavior over all nodes.

In conclusion, nodes and edges can be inactive over a long period of time. This raises the question whether the network can be treated as a static system at all. After all, edges are in fact considered as permanent in the static network. We will address this question in the next section.

### Temporal Network Analysis

In all methods described above the network was either considered as an aggregated static system or as independent snapshots. However, a closer look reveals that each snapshot is essentially not a meaningful network for disease spread, since typically *indirect* trade connections are not traversed at a single time step. For a realistic network traversal, edges at different time steps are necessary.

Thus the network under consideration is in fact a *temporal network*. Existing results and methods from classical social network analysis cannot necessarily be transferred to temporal networks. Overviews of temporal network analysis are provided in [52, 54, 55] and Chapter 4 in [56]. Analyses of epidemic spreading in temporal networks can be found in [28, 53, 57–60].

In this article, we choose a fundamental approach to analyze the pig trade network as a temporal network: the common ground between static networks and temporal networks is the accessibility matrix, i.e. the information whether a node can reach another node via an indirect connection. These connections are called paths.

Let us first consider a static network *G* = (*V*,*E*). A path *P*_*i* → *j*_ from node *i* to node *j* is formally given by a sequence of edges between these nodes where the edges can traverse arbitrary other nodes *x*_*k*_ ∈ *V*, i.e.

$$P_{i\to j}=\left[\left(i,x_{1}\right),\left(x_{1},x_{2}\right),\ldots ,\left(x_{n},j\right)\right].$$ (6)

The number of steps is the path length. In general, a large number of paths exists between each node pair in a network. For the initial spread of infectious diseases from node *i* to node *j*, only the *shortest path* is of importance, since any longer path between *i* and *j* would just correspond to a repeated infection.

In order to take the dynamic nature of trade (in particular heterogeneously distributed waiting times, Fig 11) into account, every edge of the network has to be tagged with a timestamp. A temporal network is formally given by G=(V,E), where *V* is a set of nodes and E is a set of temporal edges [54]. An edge (i,j,t)∈E connects nodes *i* and *j* at time *t*. Concerning the static paths defined in Eq (6), an important difference in temporal networks is the fact that successive edges require timestamps that are successive as well. In other words a path in a temporal network has to be *causal*. We refer to a *causal path* from node *i* to *j* as *P*_*i*⇝*j*_. Thus, it follows by analogy to Eq (6)

$$P_{i⇝j}=\left[\left(i,x_{1},t_{1}\right),\left(x_{1},x_{2},t_{2}\right),\ldots ,\left(x_{n},j,t_{n}\right)\right]$$ (7)

with the causality constraint

$$t_{1}<t_{2}<\cdots <t_{n}.$$ (8)

The *path duration* is defined as *t*_*n*_. Consequently, the shortest path duration from node *i* to *j* is that connection where *t*_*n*_ in *P*_*i*⇝*j*_ is minimal.

It is important to emphasize that due to the causality constraint, network traversal cannot be carried over from the static to the temporal case in a straightforward manner. On the other hand, the concept of *accessibility* holds also for the temporal case. Therefore, we will use this common ground in order to analyze the pig trade in terms of a temporal network.

The accessibility of a static network can be written as a matrix **P** with entries:

$${\left(P\right)}_{ij}=\left\{\begin{array}{cc} 1 & P_{i\to j}\;\text{exists}\\ 0 & \text{else}. \end{array}\right.$$ (9)

The accessibility matrix can be interpreted as the adjacency matrix of the corresponding accessibility graph. For the temporal case, the accessibility matrix P has the entries:

$${\left(P\right)}_{ij}=\left\{\begin{array}{cc} 1 & P_{i⇝j}\;\text{exists}\\ 0 & \text{else}. \end{array}\right.$$ (10)

**Fig 12:**
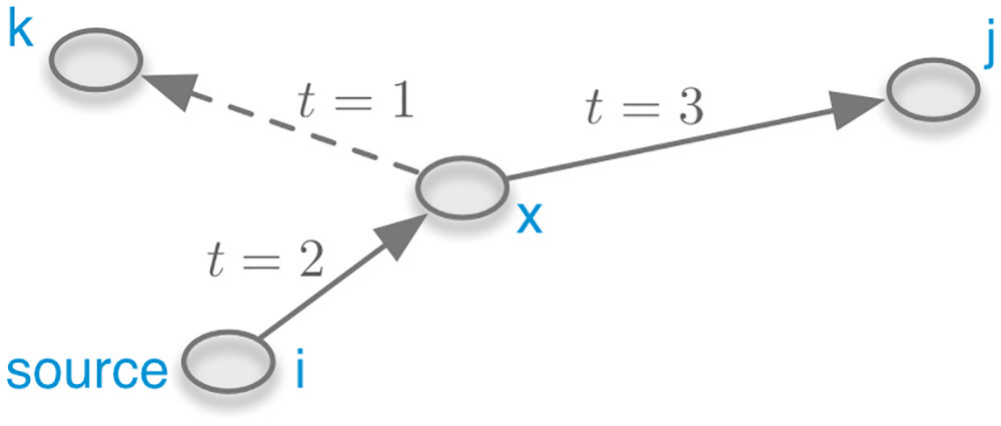
Causal path between nodes *i* and *j* in a temporal network. Although the path *P*_*i*⇝*k*_ does not exist in the temporal network, this path exists in the static case.

Fig 12 shows an exemplary causal path between nodes *i* and *j*. Although there is no causal path from *i* to *k*, this path would exist in the static view on the network. If *i* was the source node of an epidemic outbreak, the epidemic could never reach node *k* and a static view on the network would overestimate the outbreak size.

The authors would like to stress the fact that even in the temporal case the accessibility matrix represents a mathematical graph and is a static quantity. Thus, all concepts above can be transmitted one-to-one from **P** (static network) to P (temporal network).

#### Computation of the Accessibility Matrix

Given a *static* network with *N* nodes the accessibility matrix can be computed as follows [61]:

$$P∼\sum_{n}^{N}A^{n},$$ (11)

where **A** is the adjacency matrix of the network. Nevertheless, more efficient methods can be used here [62].

Given a *temporal* network of *T* time steps, the accessibility matrix as it was formally defined in Eq (10) can be computed explicitly as follows ([63, 64] and Chapter 4.3 in [56]):

$$P=\prod_{t=1}^{T}\left(A_{t}+1\right),$$ (12)

where **A**_*t*_ is a snapshot of the network at time *t* (see Eq (4)) and **1** is the identity matrix. The accessibility defined in Eq (12) takes the causality of paths into account. Following Eq P, the entries of the accessibility matrix represent the number of paths between node pairs. In most cases this number is not relevant. Thus, P can be treated as a Boolean matrix for convenience, i.e. all non zero elements are set to unity.

#### Range

As we have seen above, the accessibility matrix contains the information whether an infection started at some node *i* can reach another node *j* at all. This has been used implicitly already in Fig 4, whereby the range of a node *i* can be computed as *r*_*i*_ = ∑_*j*_(**P**)_*ij*_.

This definition can be transferred to the temporal network case in a straightforward manner. Once the accessibility matrix is computed, the (temporal) range of a node is

$$r_{i}=\sum_{j}{\left(P\right)}_{ij}.$$ (13)

In case of a disease outbreak starting at node *i*, this quantity gives the number of nodes that can potentially be infected.

**Fig 13:**
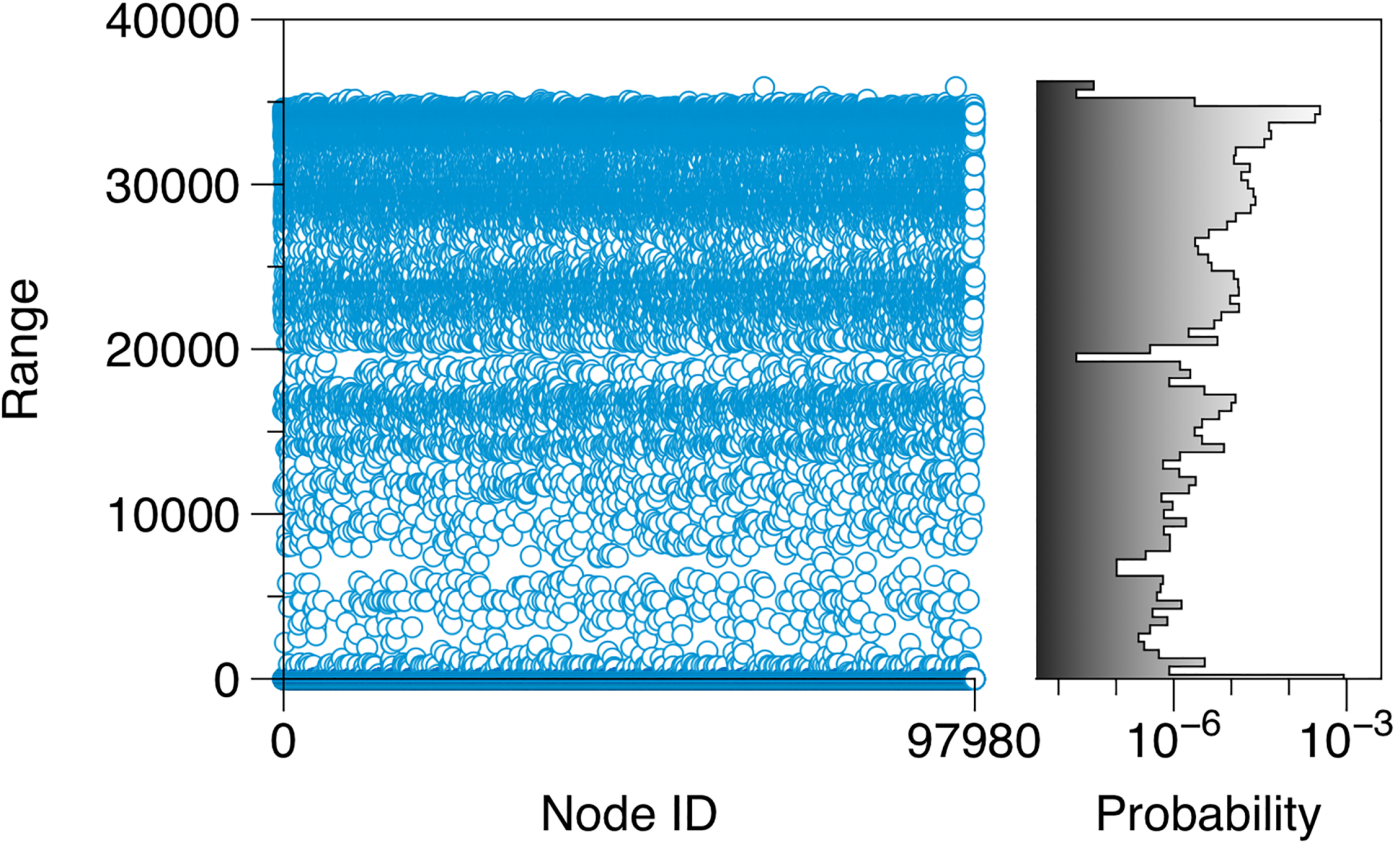
Range for every node in the temporal network. The right panel shows the histogram of the y-axis values on a log scale. In contrast to the static case (see Fig 4) the values cover the whole spectrum from minimum to maximum range.

Fig 13 shows the range for each node in the temporal pig trade network. The chart is the counterpart to Fig 4. The bimodal distribution as observed in Fig 4 is preserved also for the temporal case, although the shape is less pronounced. In contrast to the static case, temporal ranges are observed over the whole spectrum of possible values. This finding suggests that the temporal network does not contain a clear GSCC. In fact, the concept of connectedness in temporal networks is associated with some conceptional problems [54, 65].

The maximum range in the temporal network is 35,905 (for comparison: 41,369 in the static case). On average the temporal range is 17,186.8 (static: 23,154.2). In summary, the average size of an outbreak is overestimated in the static case by almost 35% and the maximum outbreak size by about 15%. It turns out here already that the analysis of this temporal network gives significantly different outbreak patterns than those observed for the static network representation. We will define the error of the static representation of a temporal network and implications for epidemiology below.

#### Path Density

The number of edges in the accessibility matrix contains important information about a network. In case of the adjacency matrix the number of nonzero elements is up to a constant the edge density of the network, i.e. *ρ*(**A**) = (∑_*ij*_(**A**)_*ij*_)/*N*(*N* − 1). Analogously, the *path density* of a (static) network is given by *ρ*(**P**) = (∑_*ij*_(**P**)_*ij*_)/*N*^2^. The factor *N*^2^ is chosen since nodes can have a path back to themselves.

For the temporal case, we define the path density:

$$\rho \left(P\right)=\frac{\sum_{ij}{\left(P\right)}_{ij}}{N^{2}}.$$ (14)

The path density takes values between 0 and 1. It should be noted that Eq (14) holds for Boolean matrices (in general $\rho \left(P\right)=\text{nnz}\left(P\right)/N^{2}$, where nnz(P) is the number of non zero elements of P). It contains the information whether a network contains structural holes: In the limit of a high path density, i.e. ρ(P)≈1, most nodes can reach each other. On the contrary, for a low path density (ρ(P)≈0) the network tends to be temporarily disconnected [65]. For the pig trade network we measure *ρ*(**P**) ≈ 0.24 for the static case (see Table 1) and ρ(P)≈0.18 for the temporal case.

#### Comparison between Static and Temporal Network Representation

The static network as described above is an approximation of the temporal system. This approximation is obtained by temporal aggregation and this means a removal of causality in paths. As stated above, causality plays an important role for the traversal of temporal networks. This raises the question to what extend a static network representation reflects the real causal accessibility between node pairs correctly.

**Fig 14:**
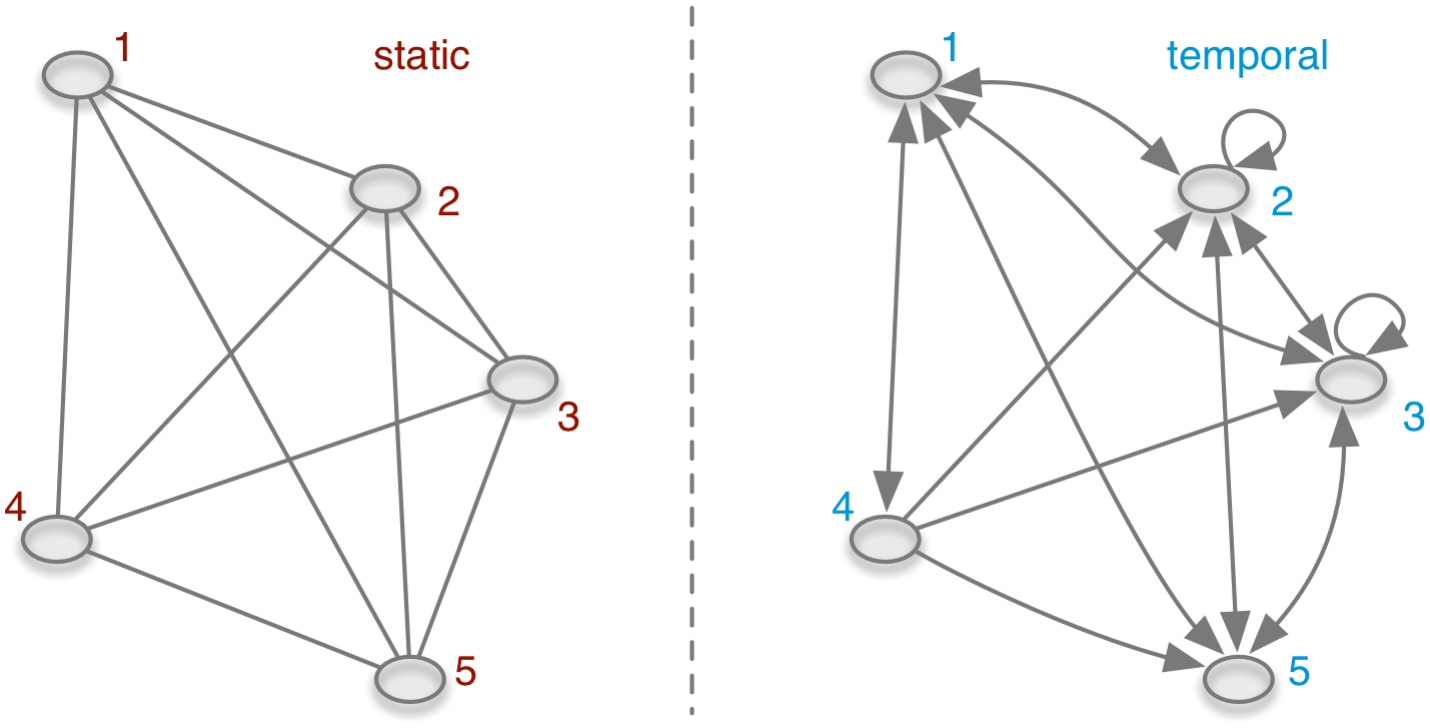
Accessibility graphs of the network of Fig 8. All nodes in the static accessibility graph (left) have a path back to themselves (i.e. self-loops, not shown). Note that although the underlying temporal network is undirected, the temporal accessibility graph is directed.

The difference between the accessibility of a temporal network and its static representation is illustrated in Fig 14. If the network is aggregated over time, a path from every node to every other node in the network would be present, i.e. it exists *P*_*i* → *j*_ for all nodes *i* and *j* including paths from a node back to itself (so-called *self-loops*). For the temporal case the following paths do not exist: *P*_2⇝4_, *P*_3⇝4_, *P*_5⇝4_ and the self-loops *P*_1⇝1_, *P*_4⇝4_ and *P*_5⇝5_. It should be noted that the consideration of self-loops is a matter of definition. In large systems (as the pig trade network) self-loops are statistically irrelevant, i.e. the number of possible self-loops is small (order of *N*) compared to the number of possible paths (order of *N*^2^). In addition, Fig 14 demonstrates an interesting feature about accessibility graphs of temporal networks: even if the underlying network is undirected, the accessibility graph of a temporal network is generally directed.

In order to quantify the error of the static representation of a temporal network, the number of paths in the static view can be compared to the number of paths in the temporal system [64]. Their ratio is called *causal fidelity* *c*, where

$$c=\frac{\sum_{ij}P_{ij}}{\sum_{ij}P_{ij}}=\frac{\rho (P)}{\rho (P)}=\frac{\text{number}\;\text{of}\;\text{paths}\;\text{in}\;G}{\text{number}\;\text{of}\;\text{paths}\;\text{in}\;G}.$$ (15)

The number of paths is the number of non zero elements in P or **P**, respectively. A causal fidelity of 1 means that a temporal network is well represented by its static counterpart. On the other hand, when *c* ≈ 0, the network should not be considered as a static system, since most paths are not causal.

In the example in Fig 14, there are mutual paths between all five nodes (including self-loops), i.e. ∑_*ij*_ **P**_*ij*_ = 5^2^ = 25. On the other hand, in the temporal case we have $\sum_{ij}P_{ij}=19$ paths. Thus, the causal fidelity is *c* = 19/25.

For the pig trade network, we measure a causal fidelity of

$$c_{\text{pig}\;\text{trade}}=\frac{1,683,966,477}{2,268,652,889}\approx 0.74.$$ (16)

This implies that 26% of the paths that appear to be present in the static network, do not actually exist. As already indicated above, the reciprocal causal fidelity gives an estimation, to what extend a static network view would overestimate a disease outbreak. Therefore, we define the *causal error* of the static network as

$$\epsilon_{\text{pig}\;\text{trade}}=\frac{1}{c_{\text{pig}\;\text{trade}}}\approx 1.35.$$ (17)

This value refers to the number of potentially infected nodes for a worst case outbreak scenario over the whole observation time. Consequently, in such a scenario a static representation of the pig trade network would overestimate the size of a disease outbreak by a factor of 1.35. It should be mentioned that the causal error is not normalized as this is the case for causal fidelity.

#### Unfolding Accessibility

In the previous section, we discussed how an accessibility graph can be computed. We hereby took into account the whole available time period. Nevertheless there is more information in the accessibility graph. In short, this information can be retrieved if the accessibility matrix is computed step by step and the path density *ρ* (see Eq (14)) is stored at every step. Hereby, we want to answer the following questions:

1. how can the dynamics of an outbreak be modeled in a temporal network?
2. what is the expected time scale of such an outbreak?

The second question aims at the fact that a temporal network exhibits not only a topological path length, but also a path *duration* (see Eq (8)). It is indeed possible that the average shortest path length of a network is short compared to the network size (see Table 1), but the path duration is very long. In other words, even a short path can take a lot of time. This information is of major interest for disease control since it provides an estimation of the time scale of a disease.

In order to answer the questions above, we consider the accessibility matrix as defined in Eq (12), but we consider *T* in Eq (12) as the evolving time *t* < *T*. Hereby, we stepwise store the current result at time *t*, i.e.

$$P\left(t\right)=\prod_{t^{\prime }=1}^{t}\left(A_{t^{\prime }}+1\right).$$ (18)

Eq (18) yields the temporal evolution of the accessibility. The process of the stepwise computation is referred to as *unfolding accessibility* [64]. Hereby, the focus is on the path *density* since it is a real number (and not a matrix).

Starting at *t* = 1, the matrix P(t) contains self-loops and the paths to the nearest neighbors. The former is a necessary artifact to allow for paths after inactive periods (see [64] for details). At *t* = 2 the matrix contains all new paths at that time step as well as all former paths and so forth. Thus, the path density grows with every time step. This process mimics an SI-type (susceptible—infected) spreading process with infection probability 1 on the network. That means every causal path is a potential route along which a disease can spread and a node is exposed, whenever it lies on such an infective path.

In analogy to the range defined above Eq (13), the current range *r*_*i*_(*t*) of a node *i* is given by

$$r_{i}\left(t\right)=\sum_{j}{\left(P\left(t\right)\right)}_{ij}.$$ (19)

**Fig 15:**
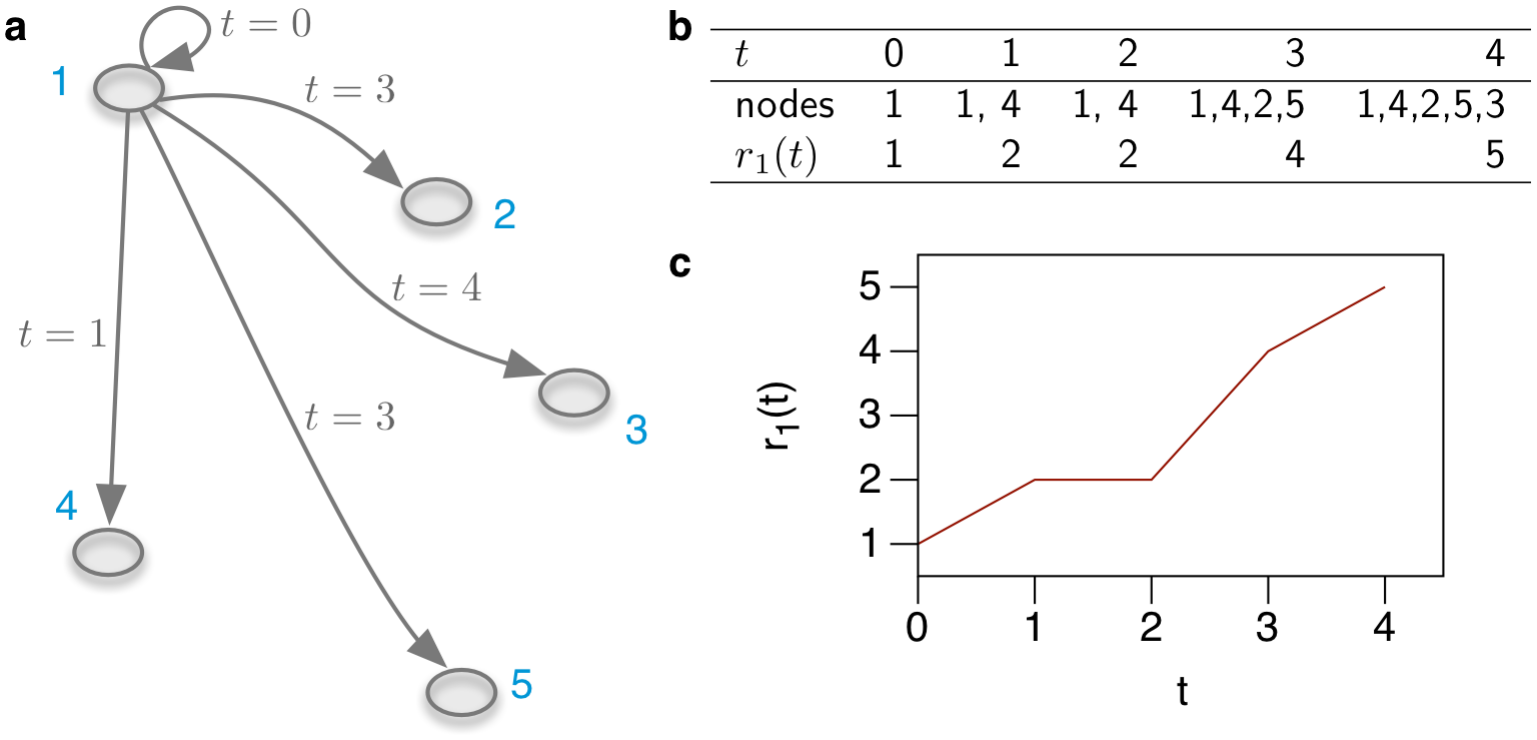
Unfolding accessibility for node 1 in the network shown in Fig 8. **a** Accessibility of node 1 including time stamps when nodes are accessed. **b** Number of infected nodes over time. **c** Infection curve (i.e. range) for source node 1.

The *herd prevalence* of an SI-process is then given by *r*_*i*_(*t*)/*N*. As an example, Fig 15 shows the current range of a node in the network from Fig 8.

In order to obtain the average herd prevalence $\bar{r}\left(t\right)/N$, one can average over all starting nodes, i.e.

$$\bar{r}\left(t\right)=\sum_{ij}{\left(P\left(t\right)\right)}_{ij}=\rho \left(P\left(t\right)\right).$$ (20)

Using Eqs (19) and (20) the first question is already answered. In short, an SI-process can be modeled by calculating the temporal evolution of the accessibility matrix. Hence, the path density at every time step corresponds to the average herd prevalence over all starting nodes.

In order to answer the second question, we have to find the distribution of path durations. Considering again the new established paths at every time step, ρ(P(t=2)) for example contains the number of new paths at time *t* = 2 *plus* the number of paths at *t* = 1 and so forth. In fact, this corresponds to the cumulative distribution of shortest path durations. Consequently, $F_{t}=\rho \left(P\left(t\right)\right)/N^{2}$ is the cumulative distribution function (CDF) of path durations in a temporal network. In this definition the cumulative distribution function is not necessarily normalized. We consider them as ‘improper’ distribution functions. The desired shortest path duration distribution is given by *dF*_*t*_/*dt*.

**Fig 16:**
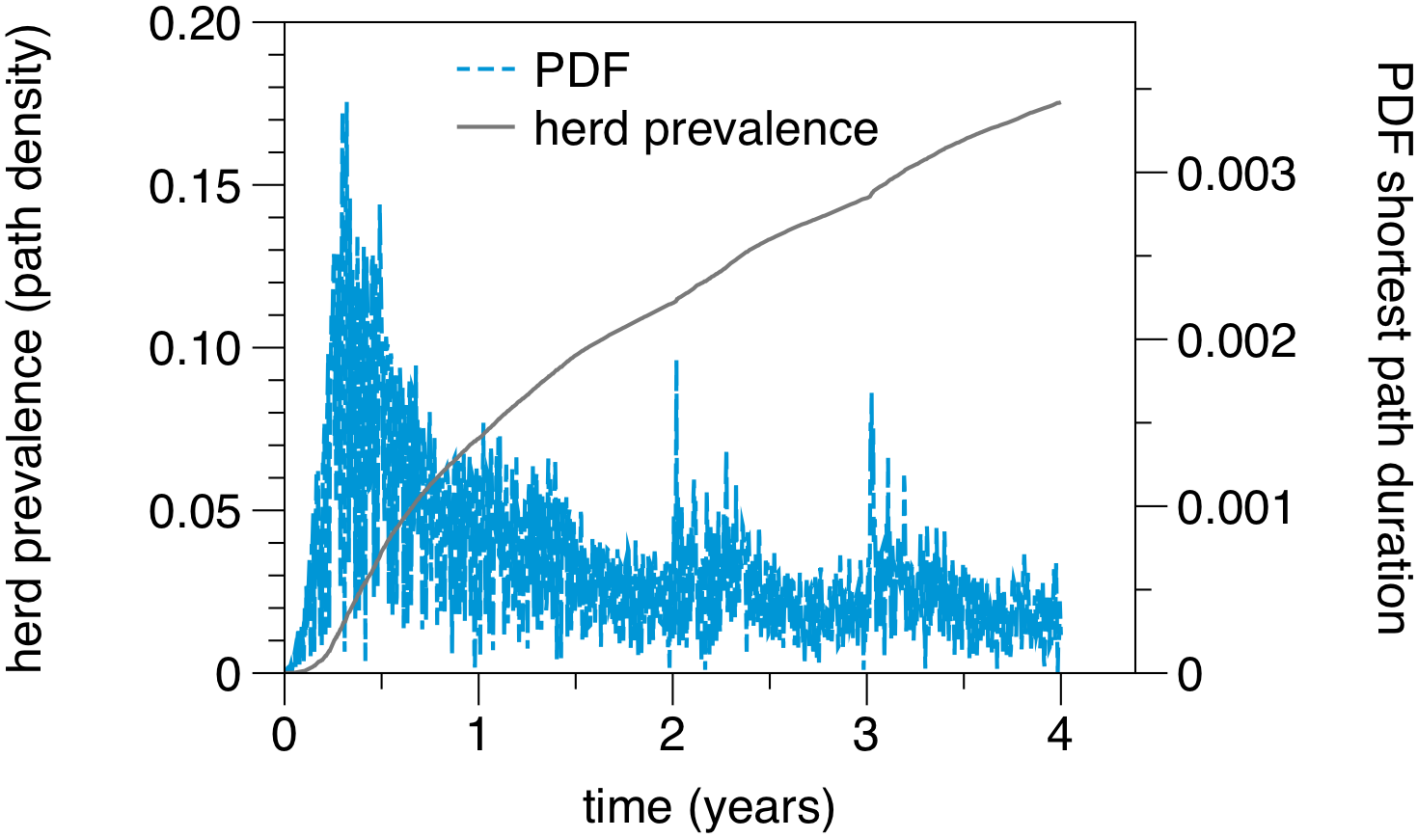
Unfolding accessibility of the temporal pig trade network. The mean herd prevalence (grey solid line) is given by the path density. The probability density function (PDF) of shortest path durations (blue dotted line) shows a global maximum at about 120 days.

Fig 16 shows the path density (grey solid line) and the probability distribution of shortest path durations (blue dashed line) of the network [64]. The path density corresponds to the mean infection curve. It shows the typical shape of an SI-infection curve, although no saturation is reached due to limited observation time in the data set. The shortest path duration (blue dotted line) shows a significant maximum at around half a year. It reaches its peak at about 120 days. This means that the majority of paths for infection spread by pig trade in Germany take 120 days. Roughly speaking, 120 days is a typical time scale for infection spread. It is important to stress the fact that this time scale does not depend on any specific disease parameters, but is a pure property of the network as a substrate for spreading. An explanation for this time scale can be found in the structure of the underlying pork production chain (see Fig 1). It defines the temporal diameter of the network (180 days). As observed in Fig 16, this diameter should limit the distribution of shortest path durations. The maximum is below that value, since there are more possible shorter paths in the production chain than the maximum (longest) path.

#### Causal Contact Tracing

The unfolding accessibility method explained above can be used in a straightforward manner for contact tracing in temporal networks. As an addition to existing contact tracing software [66], the method proposed here provides a contact tracing where concepts such as causal error and path density can be analyzed mathematically.

Tracing forward over a certain period *τ* is equivalent to an accessibility unfolding from the assumed date of entry to *t* = *τ*. Using our method, possible paths for infection are computed for all source nodes at once. However, if one is only interested in contact premises of one single index premise, the equation for unfolding accessibility Eq (18) can be rewritten accordingly. Let the infection status of the network at time *t* be given by a row-vector **x**(*t*) with *x*_*i*_ = 0, where *x*_*i*_ = 0 if node *i* is susceptible and *x*_*i*_ ≠ 0 if node *i* is infected. If node *j* is the only node infected at the starting time, then the initial vector is **x**(0) where *x*_*j*_ = 1 and *x*_*i*_ = 0 otherwise. The newly infected nodes for every time step are given by the vector

$$i\left(t+1\right)=x\left(t+1\right)-x\left(t\right)=x\left(t\right)A_{t}.$$ (21)

This equation follows immediately from Eq (18) The sequence **i**(0), **i**(1), …, **i**(*t*) represents the causal tree of possible contacts of the index node up to time *t*.

In case of a disease outbreak one is also interested in tracing backward. Therefore, the network has to be traversed backwards in time. Here we can again make use of Eq (21), but the network has to be time reversed in the first place. If a temporal network is given by a sequence of adjacency matrices Eq (4), then the time reversed network is

$$A^{-1}=A_{T}^{T},\ldots ,A_{2}^{T},A_{1}^{T},$$ (22)

where $A_{i}^{T}$ is the transposed of the *i*-th matrix in the sequence. In other words, the edge direction in each snapshot as well as the temporal ordering of the matrices is reversed. In order to realize a tracing backward, the new adjacency matrix sequence Eq (22) can be used in Eq (21).

Depending on the context, it might be reasonable to allow the traversal to have multiple edges within a single time step. This is the case if there are causal contact chains below the temporal resolution of the network (here 1*d*). As an example, a farm could buy animals from farm *i* in the morning and sell animals to farm *j* in the afternoon. The path from *i* to *j* would not be considered using the approach above. Another example is bad reporting compliance in the sense that multiple transactions might be reported for the same day, but actually happened at multiple points in time. If such circumstances have to be taken into account, we call the tracing procedure *prudent contact tracing*.

In this case, longer static paths have to be considered for every snapshot of the system [63]. Therefore, outbreaks are larger in general. For single nodes, this can have a considerable impact on the number of possible contact nodes. Nevertheless, if the network is considered as a whole, the effect is rather small and results do not change qualitatively (Section F in S1 File).

#### Can we trust the static network representation?

The found results raise the question whether a static network should be used at all as a substrate for the spread of an infectious disease. The path structure and the causal fidelity have demonstrated that for the reason of causality alone there is a discrepancy between both views. In addition, it is important to stress the fact that the concept of time does not per se exist in static networks. Due to the small average shortest path length in the static network (small world effect, Table 1), simple network traversal models of disease spread would result in unrealistic time scales. Therefore, any outbreak model on the static network requires the definition of some dynamic process, which includes the definition of parameters. The time scale of such a process might, among other things, be influenced by the network topology—for instance speed-up by degree correlations (see Section *Mixing Patterns*)—but waiting times on the nodes are not considered. However, these waiting times play a central role in the form of production times particularly for production networks, such as the pig trade network considered here. They substantially define the time scale for network traversal.

Furthermore, it should be noted that some measures defined for static networks might be defined in a more complex way (or even not at all) for the temporal case. As an example the shortest path distance in static networks has three different counterparts in temporal networks [54, 56].

Nevertheless, the static network model is certainly not redundant due to these circumstances. For many applications in veterinary medicine, centrality measures are of great importance. On static networks these measures can be easily defined, computed and interpreted. Furthermore, it has been shown that some static measures show a good correlation with those for the temporal case [28]. Hence, centrality measures computed for the static representation remain relevant also for the temporal case. In the context of risk based interventions, results from a temporal network analysis could be used in order to improve the quality of static centrality estimations.

**Fig 17:**
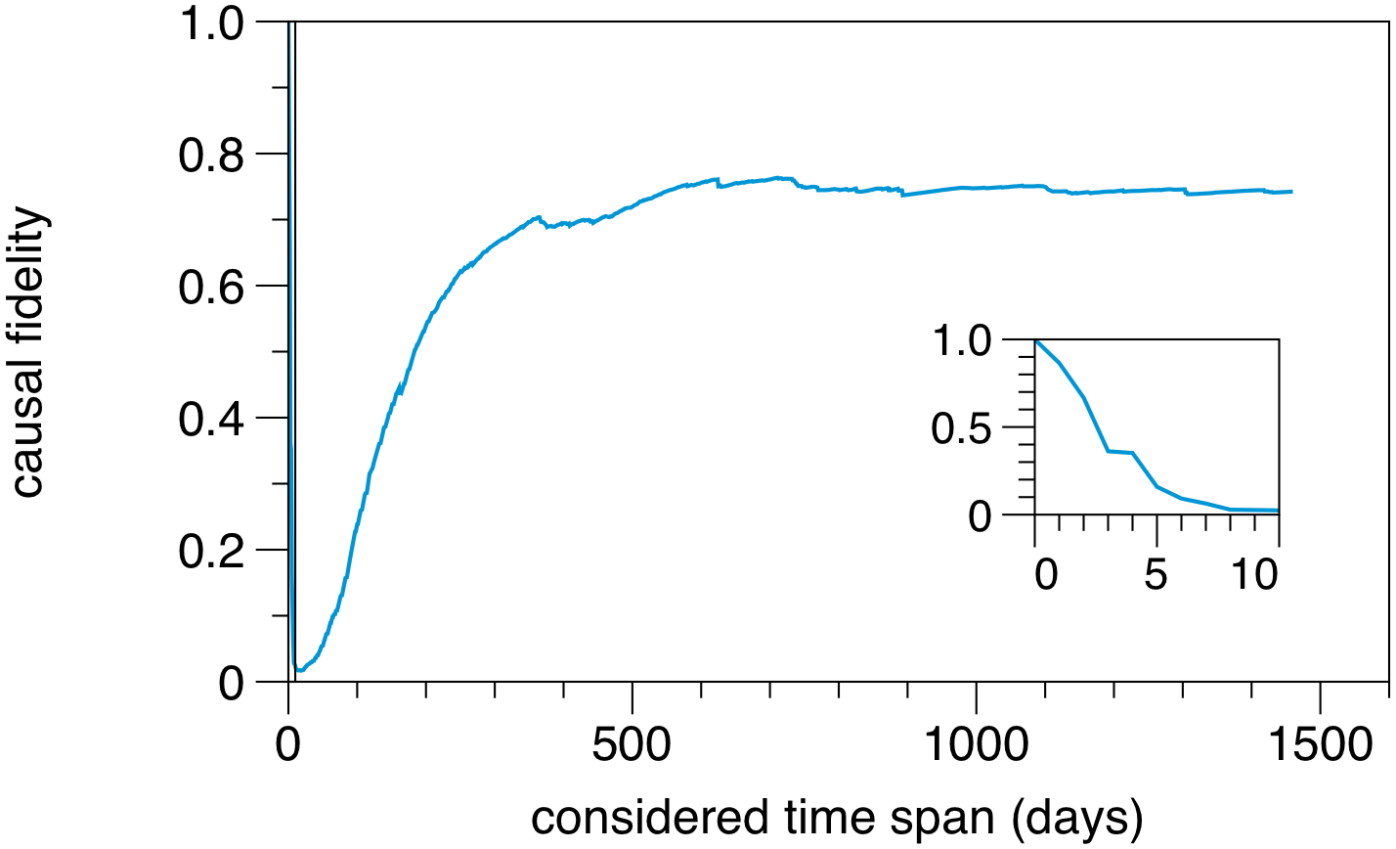
Causal fidelity for different aggregation windows. For aggregation windows < 365 days, the static network representation should be used with caution. A static network view is also adequate for very short aggregation windows (inset). The static network considered in this work corresponds to the rightmost datapoint, i.e. the maximum possible aggregation window.

Finally, we focus on the optimum aggregation window for a temporal network, such that the aggregated, static network captures causality sufficiently. Whether a network can be considered as a static one is determined by the causal fidelity in the first place. Strictly speaking the causal fidelity depends on the considered time span. This time span corresponds to the aggregation window used for the static network view. Fig 17 shows the causal fidelity for different aggregation windows. That is, every day *x* on the *x*-axis means that the dataset is considered from 01/01/2011 until 01/01/2011 + *x* days. For very short time scales (here up to 2 days) the connectivity between node pairs is provided by single edges, i.e. paths of length 1. Since causality is always maintained for paths of this length, the statistical chance for a break in the causal chain is low. Therefore, the static view performs well in this range. However, it should be noted that such a small aggregation window almost corresponds to the fully time resolved temporal network.

For intermediate time scales (2–180 days) already longer paths appear, whereby only a small number of paths exist between each node pair. This explains the low causal fidelity in this range. Between 180 and 365 days, the number of paths between each node pair increases strongly until a relatively constant causal fidelity is found for more than 365 days.

In conclusion, the static network representation provides a good picture of the real topology for very small aggregation windows (< 3 days) and for aggregation windows > 365 days. Thus, this also applies for the static network considered in this article (full aggregation window, rightmost datapoint in Fig 17). It is important to stress the fact that the mentioned small aggregation window of less than 3 days is trivial and mimics the behavior of the unaggregated network. Therefore, if the considered time scales a less then one year, results drawn from a static network representation should be treated with caution.

## Conclusion

### Summary and Discussion

It was the aim of this article to analyze the pig trade network in Germany with respect to its ability to spread infectious diseases. We have carried out this analysis avoiding the usage of external parameters. Thus, the obtained results do not depend on specific disease models. Central questions were: (1) what is the large scale structure of the system, (2) where should disease control measures be located efficiently and (3) what amount of data should be used in order to obtain an appropriate picture of possible spreading paths?

On a global scale, the directed nature of trade plays a crucial role; the network exhibits a large scale component structure, which in turn causes a sharp classification of the nodes into two risk classes. Groups of nodes having a large risk of infecting large parts of the system can be found using the ranges of the nodes.

Besides the component structure, the network has a tendency to form subgroups, where little mixing occurs between these subgroups. In particular, the federal states of Germany show such a behavior. This result suggests that it might be possible to establish zones or compartments for the German pig production. Zoning and compartmentalization are tools to define regions within a country with a certain health status to limit the trade restrictions for diseases [11] (e.g. african swine fever in Lithuania [67]). Hence, these zones/compartments might be used as a basis for a contingency plan.

Furthermore, there is a weak tendency for nodes of small degree to connect with nodes of large degree. This fact can be used to make disease control more effective, since implementing control measures (e.g. trade restrictions, culling and/or increased biosecurity measures) to large-degree nodes provides local ‘firewalls’ for all small-degree nodes attached to them. In addition, these nodes can be used for targeted surveillance in order to reduce surveillance costs. The structure of the pork production system suggests that a significant contribution to the degree mixing pattern is made by farms and slaughterhouses as well as piglet producers and other farms. However, the central part of the production chains (without slaughterhouses and piglet producers) shows this property as well. Hence, we conclude that disease control measures and surveillance can be efficiently applied in the considered network.

The efficiency of disease control measures has additionally been investigated by targeted node removal. For this purpose, different centrality measures have been computed for the nodes in order to obtain a risk based node ranking. We found that it is sufficient to remove 0.1% of the nodes in order to disassemble the network into small islands. For comparison, in the case of random node removal, 30% of the nodes would have been required to obtain the same result.

The results discussed above hardly change when the trade volume (number of traded animals) is taken into account. We conclude therefore that the relevance of the trade volume is of secondary importance in this context.

The authors would like to emphasize that a static consideration of the network shows significant shortcomings for the understanding of disease dynamics. To begin with, a static network does not contain any time scale by definition. Hence, the duration of an epidemic for example cannot be estimated from the analysis of network topology alone. Considering the network as a series of aggregated snapshots improves the results, but does not take into account causality for infectious trade paths. In addition to that, possible outbreak sizes are systematically overestimated in a static view on the network.

In order to avoid the shortcomings of the static analysis, we thereafter regarded the system as a temporal network. We thereby took into account the causal occurrences of edges explicitly. Using the unfolding accessibility method, we were able to extract temporal information about a potential epidemic outbreak. This approach mimics an SI-spreading process on the temporal network, where a worst case scenario, i.e. a transmission probability of one is assumed. It is therefore also an appropriate tool for causal contact tracing. Overall, the unfolding accessibility method provides both a large scale view on the network and can be used in order to detect central points in the network.

The most probable time for a disease to reach an arbitrary node in the network is 120 days. Even though it takes on average only 5.5 steps (see Table 1) to traverse the network, these steps take in most cases 120 days. Thus, the network shows the small world, but also the slow world property. Finally, the error of the static view on the actually temporal system can be quantified using the causal fidelity measure, which compares the possible outbreak sizes in the static and the temporal system, respectively. The causal error of the static network is approximately *ϵ* = 1.35, i.e. a static view on the network overestimates the maximum possible outbreak size by 35%.

The discrepancy found between the static and temporal network views raises the question, whether results from static network analysis can be trusted at all. As a matter of fact, a time resolved analysis is in principle superior to a static one, since it simply considers more information. Nevertheless, a number of conceptional terms known for static networks are at present not defined or much harder to interpret for the temporal case. One important example is the concept of connected components, which can be easily computed and interpreted in static networks. In a temporal network, a connected component can have multiple equally plausible meanings [54]. Conclusions such as risk classes defined by node ranges are much more difficult to interpret in temporal networks [28]. This also applies to all other centrality measures computed in temporal networks. They are significantly harder to compute and are subject to significant fluctuations, i.e. their values depend on the considered point in time. Hence, they are less suitable for control strategies, since even the node rankings are not necessarily constant over time [28]. Still a correlation between static and temporal network centrality measures could be shown in [28], and thus rankings found in static networks are still of use for pinpointing farms for targeted counter measures.

On the other hand, a consideration of temporal data is irrelevant for other applications such as mixing patterns as they could be used for zoning and compartmentalization. This is due to the fact that the required data is aggregated over many nodes and/or long time scales (e.g. trade volumes for federal states) for logical reasons.

Nevertheless, for problems like estimating outbreak time scales the usage of temporal network data is indispensable. The methods shown in this work allow for the computation of outbreak time scales with relatively little computational and conceptional effort. The same applies to tracing and the estimation of (maximum) outbreak sizes over causal paths as they can be generated from the considered data. In addition, the causal error of the static network can be computed. We have seen that this error depends on the aggregation window used to generate the static network. For the dataset considered here, the network was aggregated over a relatively long time span and we have seen that the causal error is in a stable (saturated) region. Although the static network representation still exhibits a causal inconsistency, it remains a valuable model of the pig trade system. A causal error of *ϵ* = 1.35 is still justifiable (i.e. 74% of the static paths are still present in the temporal system) and therefore a static view of the pig trade systems reflects the topological structure adequately. A temporal view should be used in particular, if network data were aggregated over smaller time spans (up to 365 days).

In conclusion, neither a static network view nor a temporal network view alone can provide the full picture of the trade system for an epidemiological application at the present time. Both methods are useful depending on the application; they are not competing theories. For decision making, measures should be used that are relatively stable over time and easy to interpret. These can be derived using static network analysis. If one is interested in realistic contact tracing or estimating epidemic time scales, a temporal network view should be used.

### Outlook

The analyzed dataset reflects trade contacts between holdings of a production network. However, the different production types of the single nodes are not resolved in the data. If these data were available, a risk assessment based on different production types could be provided as it has been done in [1]. This would significantly improve the classification of holdings into risk classes. In addition, single production chains (see Fig 1) could be reconstructed, if production type data were available. As a result, in case of an outbreak it would be possible to implement trade restrictions, while unaffected production chains remain fully functional.

The results provided here can possibly be transferred to similar production systems, but they are still computed for a particular dataset. In order to obtain a better understanding of the results found here on a more general level, more generative network [55] and dynamic [68] models could be applied.

## Supporting Information

S1 File More detailed analysis of the directed and weighted network and prudent contact tracing. A Assortativity CoefficientsB Targeted Node RemovalC Centrality in ComponentsD Weighted NetworkE Node Activity over TimeF Prudent Contact Tracing
(PDF)

## References

[1] Büttner K, Krieter J, Traulsen A, Traulsen I. Static network analysis of a pork supply chain in Northern Germany—Characterisation of the potential spread of infectious diseases via animal movements. Prev Vet Med. 2013;110(3–4):418–428. Available from: http://www.sciencedirect.com/science/article/pii/S0167587713000287. 23462679 10.1016/j.prevetmed.2013.01.008

[2] Fèvre EM, Bronsvoort BMdC, Hamilton KA, Cleaveland S. Animal movements and the spread of infectious diseases. Trends Microbiol. 2006 3;14(3):125–131. Available from: http://www.sciencedirect.com/science/article/pii/S0966842X06000175. 10.1016/j.tim.2006.01.004 16460942 PMC7119069

[3] Green DM, Kiss IZ, Kao RR. Modelling the initial spread of foot-and-mouth disease through animal movements. P Roy Soc Lond B Bio. 2006 11;273(1602):2729–2735. Available from: http://rspb.royalsocietypublishing.org/content/273/1602/2729. 10.1098/rspb.2006.3648 PMC1635508 17015320

[4] Ribbens S, Dewulf J, Koenen F, Laevens H, Kruif Ad. Transmission of classical swine fever. A review. Vet Quart. 2004 12;26(4):146–155. 10.1080/01652176.2004.9695177 15663211

[5] Thrusfield M. Veterinary epidemiology. Wiley-Blackwell; 2007.

[6] Mayer D, Reiczigel J, Rubel F. A Lagrangian particle model to predict the airborne spread of foot-and-mouth disease virus. Atmos Environ. 2008;42(3):466–479. Available from: http://www.sciencedirect.com/science/article/pii/S1352231007008643. 10.1016/j.atmosenv.2007.09.069

[7] Ducheyne E, De Deken R, Bécu S, Codina B, Nomikou K, Mangana-Vougiaki O, et al Quantifying the wind dispersal of Culicoides species in Greece and Bulgaria. Geospat Health. 2007 5;1(2):177–189. Available from: http://geospatialhealth.net/index.php/gh/article/view/266. 10.4081/gh.2007.266 18686243

[8] Martínez-López B, Ivorra B, Ramos AM, Sánchez-Vizcaíno JM. A novel spatial and stochastic model to evaluate the within- and between-farm transmission of classical swine fever virus. I. General concepts and description of the model. Vet Microbiol. 2011 1;147(3–4):300–309. 20708351 10.1016/j.vetmic.2010.07.009

[9] Olofsson E, Nöremark M, Lewerin SS. Patterns of between-farm contacts via professionals in Sweden. Acta Vet Scand. 2014;56(1):70–13. Available from: http://www.actavetscand.com/content/56/1/70. 10.1186/s13028-014-0070-2 25366065 PMC4222379

[10] Wang Y, Jin Z, Yang Z, Zhang ZK, Zhou T, Sun GQ. Global analysis of an SIS model with an infective vector on complex networks. Nonlinear Anal Real. 2012 01;13(2):543–557. Available from: http://www.sciencedirect.com/science/article/pii/S1468121811001945. 10.1016/j.nonrwa.2011.07.033

[11] OiE. Terrestrial Animal Health Code. 24th ed OiE; 2015.

[12] Knight-Jones TJD, Rushton J. The economic impacts of foot and mouth disease—What are they, how big are they and where do they occur? Preventive Veterinary Medicine. 2013;112(3–4):161–173. Available from: http://www.sciencedirect.com/science/article/pii/S0167587713002390. 10.1016/j.prevetmed.2013.07.013 23958457 PMC3989032

[13] Fritzemeier J. Epidemiology of classical swine fever in Germany in the 1990s. Vet Microbiol. 2000 11;77(1–2):29–41. Available from: http://linkinghub.elsevier.com/retrieve/pii/S0378113500002546. 10.1016/S0378-1135(00)00254-6 11042398

[14] FAO—Economic and Social Development Department. FAO [website]; 2016 [cited 2016]. Available from: http://faostat.fao.org/.

[15] Federal Ministry of Food; Agriculture (BMEL). Understanding Farming—Facts and figures about German farming. BMEL; 2015. Available from: http://www.bmel.de/SharedDocs/Downloads/EN/Publications/UnderstandingFarming.html;jsessionid=45E09F8A934E452B87D762698DBE305C.2_cid296 [cited 2015].

[16] Martínez-López B, Perez AM, Sánchez-Vizcaíno JM. Social Network Analysis. Review of General Concepts and Use in Preventive Veterinary Medicine. Transbound Emerg Dis. 2009;56:109–120. 10.1111/j.1865-1682.2009.01073.x 19341388

[17] Dubé C, Ribble C, Kelton D, Mcnab B. A Review of Network Analysis Terminology and its Application to Foot-and-Mouth Disease Modelling and Policy Development. Transbound Emerg Dis. 2009 4;56(3):73–85. 10.1111/j.1865-1682.2008.01064.x 19267879

[18] Bigras-Poulin M, Barfod K, Mortensen S, Greiner M. Relationship of trade patterns of the Danish swine industry animal movements network to potential disease spread. Prev Vet Med. 2007 7;80(2–3):143–165. Available from: http://www.sciencedirect.com/science/article/pii/S0167587707000384. 10.1016/j.prevetmed.2007.02.004 17383759

[19] Bigras-Poulin M, Thompson RA, Chriel M, Mortensen S, Greiner M. Network analysis of Danish cattle industry trade patterns as an evaluation of risk potential for disease spread. Prev Vet Med. 2006 9;76(1–2):11–39. Available from: http://www.sciencedirect.com/science/article/pii/S0167587706000778. 10.1016/j.prevetmed.2006.04.004 16780975

[20] Christley R, Robinson SE, Lysons R, French NP. Network analysis of cattle movement in Great Britain. In: Proc. Soc. Vet. Epidemiol. Prev. Med.; 2005. p. 234–244.

[21] Dutta BL, Ezanno P, Vergu E. Characteristics of the spatio-temporal network of cattle movements in France over a 5-year period. Prev Vet Med. 2014 11;117(1):79–94. 10.1016/j.prevetmed.2014.09.005 25287322

[22] Rautureau S, Dufour B, Durand B. Structural vulnerability of the French swine industry trade network to the spread of infectious diseases. Animal. 2012 7;6:1152–1162. Available from: http://journals.cambridge.org/article_S1751731111002631. 10.1017/S1751731111002631 23031477

[23] Webb CR. Farm animal networks: unraveling the contact structure of the British sheep population. Prev Vet Med. 2005 4;68(1):3–17. Available from: http://linkinghub.elsevier.com/retrieve/pii/S0167587705000073. 10.1016/j.prevetmed.2005.01.003 15795012

[24] Valdano E, Poletto C, Giovannini A, Palma D, Savini L, Colizza V. Predicting Epidemic Risk from Past Temporal Contact Data. PLoS Comput Biol. 2015 3;11(3):e1004152 Available from: http://dx.plos.org/10.1371/journal.pcbi.1004152. 10.1371/journal.pcbi.1004152 25763816 PMC4357450

[25] Natale F, Giovannini A, Savini L, Palma D, Possenti L, Fiore G, et al Network analysis of Italian cattle trade patterns and evaluation of risks for potential disease spread. Prev Vet Med. 2009 1;92:341–350. 10.1016/j.prevetmed.2009.08.026 19775765

[26] Natale F, Savini L, Giovannini A, Calistri P, Candeloro L, Fiore G. Evaluation of risk and vulnerability using a Disease Flow Centrality measure in dynamic cattle trade networks. Prev Vet Med. 2011 2;98(2–3):111–118. 10.1016/j.prevetmed.2010.11.013 21159393

[27] Stärk KDC, Regula G, Hernandez J, Knopf L, Fuchs K, Morris RS, et al Concepts for risk-based surveillance in the field of veterinary medicine and veterinary public health: review of current approaches. BMC Health Serv Res. 2006;6:20 Available from: http://eutils.ncbi.nlm.nih.gov/entrez/eutils/elink.fcgi?dbfrom=pubmed&id=16507106&retmode=ref&cmd=prlinks. 10.1186/1472-6963-6-20 16507106 PMC1409776

[28] Konschake M, Lentz HHK, Conraths FJ, Hövel P, Selhorst T. On the Robustness of In- and Out-Components in a Temporal Network. PLOS ONE. 2013 2;8(2):e55223 Available from: http://dx.plos.org/10.1371/journal.pone.0055223.s003. 10.1371/journal.pone.0055223 23405124 PMC3566222

[29] Bayerisches Staatsministerium für Ernährung, Landwirtschaft und Forsten (StMELF). Herkunftssicherungs und Informationssystem für Tiere [website]; 2016 [cited 2016]. Available from: www.hi-tier.de.

[30] Albert R, Barabási AL. Statistical mechanics of complex networks. Rev Mod Phys. 2002 1;74:47–97. Available from: http://link.aps.org/doi/10.1103/RevModPhys.74.47. 10.1103/RevModPhys.74.47

[31] Dorogovtsev S, Mendes J, Samukhin A. Giant strongly connected component of directed networks. Phys Rev E. 2001 7;64:025101(R). Available from: http://link.aps.org/doi/10.1103/PhysRevE.64.025101. 10.1103/PhysRevE.64.025101 11497638

[32] Newman MEJ. The Structure and Function of Complex Networks. SIAM Rev. 2003;45(2):167–256. 10.1137/S003614450342480

[33] Lentz HHK, Selhorst T, Sokolov IM. Spread of infectious diseases in directed and modular metapopulation networks. Phys Rev E. 2012 6;85:066111 Available from: http://link.aps.org/doi/10.1103/PhysRevE.85.066111. 10.1103/PhysRevE.85.066111 23005166

[34] Newman MEJ. Mixing patterns in networks. Phys Rev E. 2003 1;67:026126 Available from: http://link.aps.org/doi/10.1103/PhysRevE.67.026126. 10.1103/PhysRevE.67.026126 12636767

[35] Lentz HHK, Konschake M, Teske K, Kasper M, Rother B, Carmanns R, et al Trade communities and their spatial patterns in the German pork production network. Prev Vet Med. 2011;98(2–3):176–181. 10.1016/j.prevetmed.2010.10.011 21111498

[36] Holten D. Hierarchical Edge Bundles: Visualization of Adjacency Relations in Hierarchical Data. Visualization and Computer Graphics, IEEE Transactions on. 2006 9;12(5):741–748. 10.1109/TVCG.2006.147 17080795

[37] Newman MEJ. Assortative Mixing in Networks. Phys Rev Lett. 2002 1;89(20):208701 Available from: http://link.aps.org/doi/10.1103/PhysRevLett.89.208701. 10.1103/PhysRevLett.89.208701 12443515

[38] European Commission. Approved establishments—Lists of approved food establishmentsslide [website]; 2016 [cited 2016]. Available from: http://ec.europa.eu/food/food/biosafety/establishments/list_en.htm.

[39] Bundesministerium für Ernährung und Landwirtschaft (Referat 123). Statistisches Jahrbuch über Ernährung, Landwirtschaft und Forsten der Bundesrepublik Deutschland. Münster-Hiltrup: Landwirtschaftsverl.; 2014.

[40] Pastor-Satorras R, Vespignani A. Epidemic dynamics in finite size scale-free networks. Phys Rev E. 2002 3;65:035108(R). Available from: http://link.aps.org/doi/10.1103/PhysRevE.65.035108. 10.1103/PhysRevE.65.035108 11909143

[41] Pastor-Satorras R, Vespignani A. Epidemic dynamics and endemic states in complex networks. Phys Rev E. 2001 5;63:066117 Available from: http://link.aps.org/doi/10.1103/PhysRevE.63.066117. 10.1103/PhysRevE.63.066117 11415183

[42] Albert R, Jeong H, Barabási AL. Error and attack tolerance of complex networks. Nature. 2000;406(6794):378–382. 10.1038/35019019 10935628

[43] Danon L, Ford AP, House TA, Jewell CP, Keeling MJ, Roberts GO, et al Networks and the epidemiology of infectious disease. Interdiscip Perspect Infect Dis. 2011;2011:284909 Available from: http://eutils.ncbi.nlm.nih.gov/entrez/eutils/elink.fcgi?dbfrom=pubmed&id=21437001&retmode=ref&cmd=prlinks. 10.1155/2011/284909 21437001 PMC3062985

[44] Lloyd AL, May RM. Epidemiology. How viruses spread among computers and people. Science. 2001 5;292(5520):1316–1317. Available from: http://eutils.ncbi.nlm.nih.gov/entrez/eutils/elink.fcgi?dbfrom=pubmed&id=11360990&retmode=ref&cmd=prlinks. 10.1126/science.1061076 11360990

[45] Jones JH, Handcock M. Social networks: Sexual contacts and epidemic thresholds. Nature. 2003 1;423:605 Available from: http://www.ncbi.nlm.nih.gov/pubmed/12789329. 10.1038/423605a 12789329

[46] Kao RR, Danon L, Green DM, Kiss IZ. Demographic structure and pathogen dynamics on the network of livestock movements in Great Britain. Proc R Soc B. 2006;273(1597):1999–2007. Available from: http://eutils.ncbi.nlm.nih.gov/entrez/eutils/elink.fcgi?dbfrom=pubmed&id=16846906&retmode=ref&cmd=prlinks. 10.1098/rspb.2006.3505 16846906 PMC1635475

[47] Schwartz N, Cohen R, ben Avraham D, Barabási AL, Havlin S. Percolation in directed scale-free networks. Phys Rev E. 2002 7;66(1). Available from: http://link.aps.org/doi/10.1103/PhysRevE.66.015104. 10.1103/PhysRevE.66.015104 12241410

[48] Meyers L, Pourbohloul B, Newman MEJ. Network theory and SARS: predicting outbreak diversity. J Theor Biol. 2005 1;232:71–81. Available from: http://linkinghub.elsevier.com/retrieve/pii/S0022519304003510. 10.1016/j.jtbi.2004.07.026 15498594 PMC7094100

[49] Clauset A, Newman MEJ. Power-Law Distributions in Empirical Data. SIAM Rev. 2009;51(4):661 Available from: http://link.aip.org/link/SIREAD/v51/i4/p661/s1&Agg=doi. 10.1137/070710111

[50] Holme P, Kim BJ, Yoon CN, Han SK. Attack vulnerability of complex networks. Phys Rev E. 2002 5;65(5):056109 Available from: http://link.aps.org/doi/10.1103/PhysRevE.65.056109. 10.1103/PhysRevE.65.056109 12059649

[51] Pan RK, Saramäki J. Path lengths, correlations, and centrality in temporal networks. Phys Rev E. 2011 7;84(1):016105 Available from: http://link.aps.org/doi/10.1103/PhysRevE.84.016105. 10.1103/PhysRevE.84.016105 21867255

[52] Holme P, Saramäki J. Temporal networks. Phys Rep. 2012;519(3):97–125. Available from: http://www.sciencedirect.com/science/article/pii/S0370157312000841. 10.1016/j.physrep.2012.03.001

[53] Bajardi P, Barrat A, Natale F, Savini L, Colizza V. Dynamical patterns of cattle trade movements. PLOS ONE. 2011;6(5):e19869 Available from: http://eutils.ncbi.nlm.nih.gov/entrez/eutils/elink.fcgi?dbfrom=pubmed&id=21625633&retmode=ref&cmd=prlinks. 10.1371/journal.pone.0019869 21625633 PMC3097215

[54] Casteigts A, Flocchini P, Quattrociocchi W, Santoro N. Time-varying graphs and dynamic networks. Int J Parallel Emergent Distrib Syst. 2012;27(5):387–408. Available from: http://www.tandfonline.com/doi/abs/10.1080/17445760.2012.668546. 10.1080/17445760.2012.668546

[55] Holme P. Modern temporal network theory: a colloquium. Eur Phys J B. 2015 9;88(9):234–30. Available from: http://link.springer.com/10.1140/epjb/e2015-60657-4. 10.1140/epjb/e2015-60657-4

[56] Lentz HHK. Paths for epidemics in static and temporal networks [PhD Thesis]. Dissertation, Humboldt-University of Berlin; 2013. Available from: urn:nbn:de:kobv:11-100213397.

[57] Rocha LEC, Liljeros F, Holme P. Simulated Epidemics in an Empirical Spatiotemporal Network of 50,185 Sexual Contacts. PLoS Comput Biol. 2011 3;7(3):e1001109 10.1371/journal.pcbi.1001109 21445228 PMC3060161

[58] Valdano E, Ferreri L, Poletto C, Colizza V. Analytical Computation of the Epidemic Threshold on Temporal Networks. Phys Rev X. 2015 4;5(2):021005–9. Available from: http://link.aps.org/doi/10.1103/PhysRevX.5.021005.

[59] Bajardi P, Barrat A, Savini L, Colizza V. Optimizing surveillance for livestock disease spreading through animal movements. J R Soc Interface. 2012;Available from: http://rsif.royalsocietypublishing.org/content/early/2012/06/21/rsif.2012.0289.abstract. 10.1098/rsif.2012.0289 22728387 PMC3479905

[60] Vernon MC, Keeling MJ. Representing the UK’s cattle herd as static and dynamic networks. Proc R Soc B. 2009 2;276(1656):469–476. Available from: http://rspb.royalsocietypublishing.org/cgi/doi/10.1098/rspb.2008.1009. 10.1098/rspb.2008.1009 18854300 PMC2592553

[61] Warshall S. A theorem on boolean matrices. J ACM. 1962;9(1):11–12. Available from: http://portal.acm.org/citation.cfm?id=321105.321107. 10.1145/321105.321107

[62] Skiena SS. The Algorithm Design Manual. 2nd ed Springer Publishing Company, Incorporated; 2008.

[63] Grindrod P, Parsons M, Higham D, Estrada E. Communicability across evolving networks. Phys Rev E. 2011 4;83(4):046120 Available from: http://link.aps.org/doi/10.1103/PhysRevE.83.046120. 10.1103/PhysRevE.83.046120 21599253

[64] Lentz HHK, Selhorst T, Sokolov IM. Unfolding Accessibility Provides a Macroscopic Approach to Temporal Networks. Phys Rev Lett. 2013 3;110(11):118701 Available from: http://link.aps.org/doi/10.1103/PhysRevLett.110.118701. 10.1103/PhysRevLett.110.118701 25166583

[65] Nicosia V, Tang J, Musolesi M, Russo G, Mascolo C, Latora V. Components in time-varying graphs. Chaos. 2012 6;22(2):023101 Available from: http://eutils.ncbi.nlm.nih.gov/entrez/eutils/elink.fcgi?dbfrom=pubmed&id=22757508&retmode=ref&cmd=prlinks. 10.1063/1.3697996 22757508

[66] Nöremark M, Widgren S. EpiContactTrace: an R-package for contact tracing during livestock disease outbreaks and for risk-based surveillance. BMC Vet Res. 2014;10:71 Available from: http://eutils.ncbi.nlm.nih.gov/entrez/eutils/elink.fcgi?dbfrom=pubmed&id=24636731&retmode=ref&cmd=prlinks. 10.1186/1746-6148-10-71 24636731 PMC3974595

[67] EUR-Lex. Commission Implementing Decision (EU) 2015/2433 of 18 December 2015 amending Implementing Decision 2014/709/EU as regards the animal health control measures relating to African swine fever in certain Member States. European Commission; 2015. Available from: http://eur-lex.europa.eu.

[68] Wang Y, Jin Z. Global analysis of multiple routes of disease transmission on heterogeneous networks. Physica A: Statistical Mechanics and its Applications. 2013;392(18):3869–3880. Available from: http://www.sciencedirect.com/science/article/pii/S0378437113002756. 10.1016/j.physa.2013.03.042

