# Supplementary material for: Disease Spread through Animal Movements: A Static and Temporal Network Analysis of Pig Trade in Germany
Source: PLoS One. 2016 May 6;11(5):e0155196. doi: 10.1371/journal.pone.0155196 (PMC4859575; doi:10.1371/journal.pone.0155196)
Supplement: S1 File — A Assortativity CoefficientsB Targeted Node RemovalC Centrality in ComponentsD Weighted NetworkE Node Activity over TimeF Prudent Contact Tracing (PDF) [file pone.0155196.s001.pdf]

Supporting Information – Disease spread through animal movements: a static and temporal network analysis of pig trade in Germany

Lentz et al.

A Assortativity Coefficients

In Table A1 we show the assortativity coefficients for the directed *unweighted* network. The coefficients were calculated for all combinations between in-degree and out-degree. For instance in-degree – out-degree correlation means the tendency that a node of high in-degree has a directed link to a node of high out-degree. We find that the directed network is still weakly disassortative with respect to the degree. The relatively high value for out-degree – in-degree correlations maybe explained by the fact that many small premises trade to nodes of large in-degree, i.e. slaughterhouses or traders.

Table A1: *Degree assortativity coefficients for the directed unweighted network.*

Correlation	r
in-degree – in-degree	-0.086
in-degree – out-degree	-0.063
out-degree – in-degree	-0.13
out-degree – out-degree	-0.09

B Targeted Node Removal

In addition to the results in the main text, we compute the size of the GSCC after removing nodes with respect to the following centrality measures:

eigenvector centrality. C_E – Correlates with the probability that a node is visited in a random walk on the network.

pagerank. C_P – similar to C_E, but links between arbitrary nodes are possible with a small probability.

Katz centrality. C_K – Ability of a node to have shortest path to other nodes, where shorter paths have a stronger weight.

Figure B1 shows the impact of centrality based node removal for all considered centrality measures. Although node removal based on eigenvector-centrality, Katz-Centrality or Pagerank still performs better than random node removal, degree betweenness and closeness are more appropriate measures for targeted node removal.

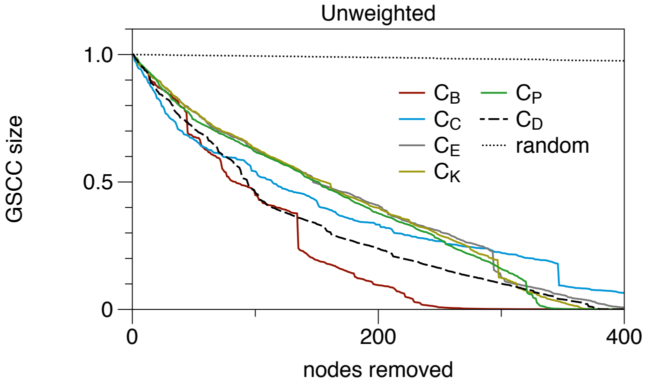

Figure B1: *Impact of centrality based node removal, when up to 1 % of the nodes are removed.* C_B*-Betweenness,* C_C*-Closeness,* C_E*-Eigenvector,* C_K*-Katz-Centrality,* C_P*-Pagerank. Size of giant strongly connected component is normalized to unity.*

C Centrality in Components

In Figure C1 we show the centrality of each node resolved by its giant component membership. Panel a) demonstrates that nodes in the GIC and GSCC have a long range. On the other hand, these nodes have a low reachability. The reachability of a node is the number of nodes that can reach that node, i.e. its range in the reversed network. Panel b) shows that many nodes of high out-degree can be found in the

GIC and many high in-degree nodes are located in the GOC. Furthermore, the correlation between in-degree and out-degree is relatively high in the GSCC. The GIC and GSCC also contain the nodes of high closeness (Panel c)). As expected, nodes with high betweenness are located on the GSCC.

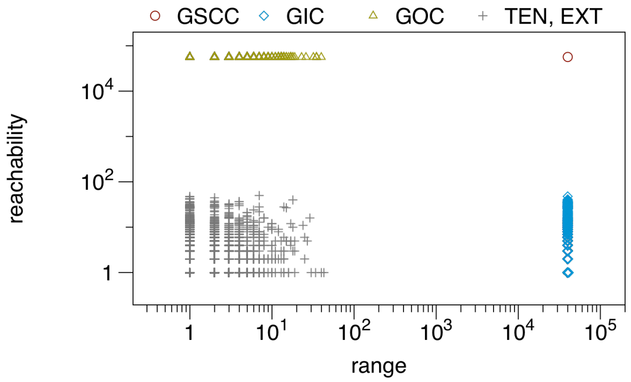

(a) *Reachability vs. range.*

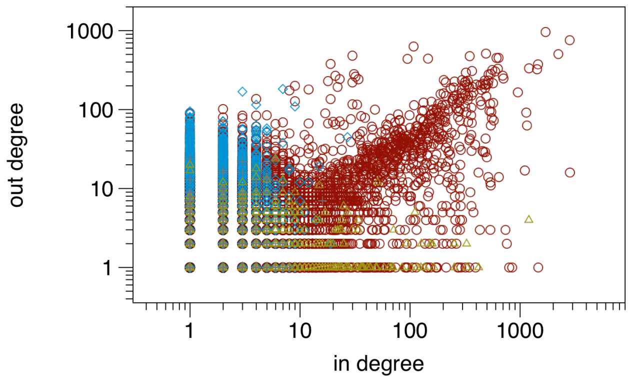

(b) *Out-degree vs. in-degree.*

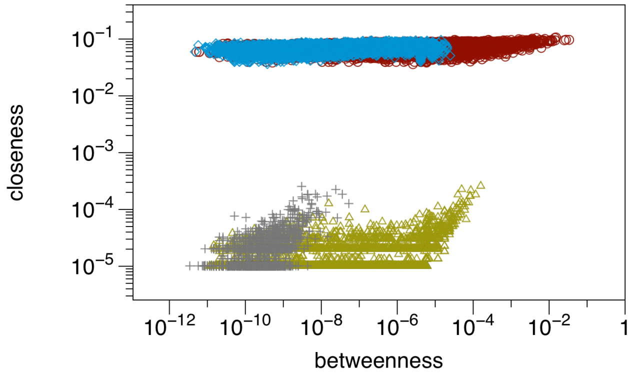

(c) *Closeness vs. betweenness.*

Figure C1: *centrality measures for different giant clusters.*

D Weighted Network

In this section we reproduce the results of the static network in the main text, but take the edge weights into account. We weight the edges of the network according to the number of traded animals.

First we focus on the large scale structure of the weighted network. For the component structure, the edge weight does not play any role, since component structure is a purely topological property of the system. The average shortest path distance is computed using Dijkstra's algorithm in the weighted network. We find the average shortest path length to be 9.7 and the diameter (longest shortest path length) to be 30. This implies that weighted shortest paths are on average twice as long as unweighted shortest paths.

The assortativity coefficients for the weighted network are shown in Table D1. Mixing coefficients for federal state, district and municipality are only marginally influenced by edge weight. Also the results about the dominant federal states remain similar to the unweighted case: Inter-state links are mainly formed between North Rhine-Westphalia (NW) and Lower Saxony (NI) as well as Bavaria (BY) and Baden-Wuerttemberg (BW). These links make up 38 % of the total trade volume. The trade between NW and NI alone accounts for 29 % of all inter-state trade connections. Concerning the Pareto-principle, 19.3 % of the weighted edges make up 80.7 % of all trade volume.

We find that the weighted degree correlations take similar values for all combinations of in-degree and out-degree. This reflects the fact independent of the combination of degrees, trade is balanced for each pair of premises.

Table D1: *Assortativity coefficients between different categories for the weighted network.*

**Correlation**	r
Federal state	0.75
District	0.38
Municipality	0.15
degree – degree	-0.050
in-degree – in-degree	-0.041
in-degree – out-degree	-0.043
out-degree – in-degree	-0.040
out-degree – out-degree	-0.042

We now focus on the microscopic structure of the weighted network. Edge weights can be used in order to compute node centrality more accurately. The weighted degree distribution shows a similar shape as for the unweighted case. Figure D2 shows the edge weight distribution of the network. Weight is

measured in terms of total number of traded animals during the observation period.

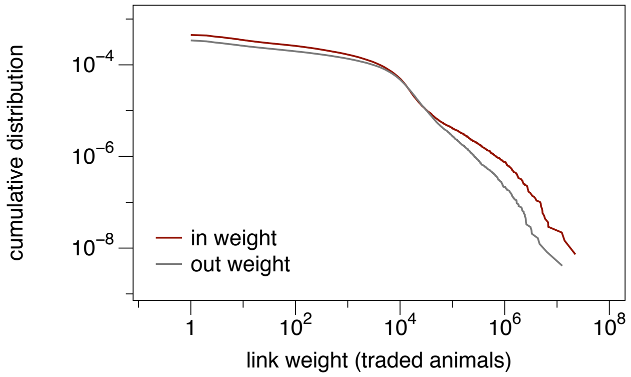

Figure D2: *Edge weight distribution.*

The edge weight plays a significant role for the computation of shortest paths as it is implicitly contained in most centrality measures. If for instance 1000 animals have been traded from node i to node j and 10 animals have been traded between nodes i and k, the weight of the edge (i, j) is significantly higher and this edge would probably be traversed in a shortest path.

We compute the centrality measures as in the main text for the weighted network. Figure D3 shows the impact of node removal based on weighted centrality measures. The results show qualitatively the same behavior as for the unweighted case.

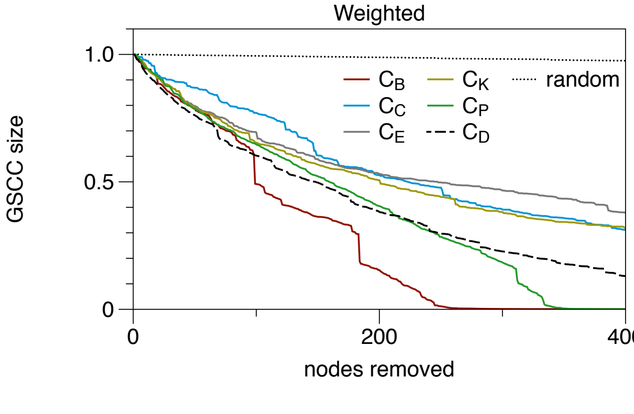

Figure D3: *Impact of centrality based node removal in the weighted network.*

Overall we obtain a similar picture as for the unweighted case: Nodes of large degree (i.e. sum of trade volume to neighbors) or betweenness perform well for targeted intervention measures. It is remarkable however that the degree shows a good performance, when only relatively few nodes are removed. Closeness performs significantly worse than in the unweighted case.

We conclude that:

1. any centrality based intervention performs significantly better than random intervention!

2. removal of high weight nodes is efficient for removal of up to 100 nodes.

3. removal of high betweenness nodes is efficient for removal of more than 100 nodes.

4. the average edge weight corresponds to a very high infection probability per edge.

Edge Weight vs Infection Probability. Finally, we estimate how edge weights can be mapped onto infection probabilities. Every transport of one or more infectious animals is equally infectious.

First, we compute the probability that *exactly* one animal is infectious $P(X = 1)$ for a transport going from farm i to j and w animals are transported. This probability is given by a binomial distribution $P(X = 1) = b_{w,p}(X = 1)$, where p is the probability that an animal is infected in the source node i. This probability is given by the prevalence in node i.

Second, we compute the probability that *at least* one animal is infectious $P(X \geq 1)$ for a transport going from farm i to j. The probability is given by $P(X \geq 1) = B_{w,p}(X = 1)$, where $B_{w,p}(X)$ is the complementary cumulative distribution function (CCDF) of $b_{w,p}(X)$. The in-farm prevalence for diseases relevant here (classical swine fever, Aujeszky's disease, foot and mouth disease) is typically 30-50 % at the time of detection [2].

For the data set considered here we observe an average edge weight of $\bar{w} \approx 100$ for every trade transaction (considering the aggregated edge weight would give $\bar{w}_{\text{total}} \approx 2000$). Assuming that the prevalence in the source farm is 30 % as explained above, i.e. $\bar{p} = 0.3$, it follows that the probability that an average trade link is infective is given by

$$P(X \geq 1) = B_{\bar{w},\bar{p}}(X = 1) \approx (100 - 10^{-14})\ \% \approx 1, \tag{1}$$

where $B_{\bar{w},\bar{p}}(X)$ is the complementary cumulative distribution function of the binomial distribution. This means that the expected infection probability per trade transaction is almost 1. Consequently, the probability of infection if significantly smaller than 1 only for low volume trade transactions.

E Node Activity over Time

Figure E1 shows the fraction of active nodes over time for two aggregation windows. For the 84 d aggregation window, the annual loss is approximately 2,800 nodes.

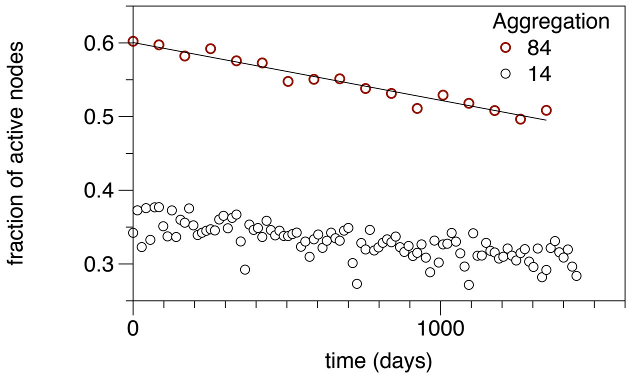

Figure E1: *Development of the node activity over the observation period.*

F Prudent Contact Tracing

In the tracing procedure mentioned in the main text a pathogen is assumed to only take one step at each snapshot. There might be necessity for some pathogens that multiple steps are allowed in every snapshot. In the context of contact tracing, we refer to this circumstance as *prudent* contact tracing. This corresponds to the situation that a pathogen goes from node i to node j and then from node j to node k and so forth at the same day. In order to take this into account, we add allow for arbitrary long paths in each snapshot [1]. Given a temporal network as a sequence of adjacency matrices $\mathcal{A} = \mathbf{A}_1, \mathbf{A}_2, \ldots, \mathbf{A}_T$, we define the long path corrected network as

$$\mathcal{B} = \sum_{i=1}^{D} \mathbf{A}_1^i, \sum_{i=1}^{D} \mathbf{A}_2^i, \ldots, \sum_{i=1}^{D} \mathbf{A}_T^i, \qquad (2)$$

where D is the diameter of the aggregated network. The measured value for the diameter is $D = 18$ (see main text). Thus, we allow for a maximum of 18 steps in each snapshot. This value can be revised downwards depending on assumptions about the disease under consideration or data quality. Given the path corrected network, prudent contact tracing can be done using the method as described in the main text, but with the temporal network as defined in (2).

Figure F1 shows the range of a node using the standard approach vs. the prudent range computed using (2). The deviation between the two is 148 on average.

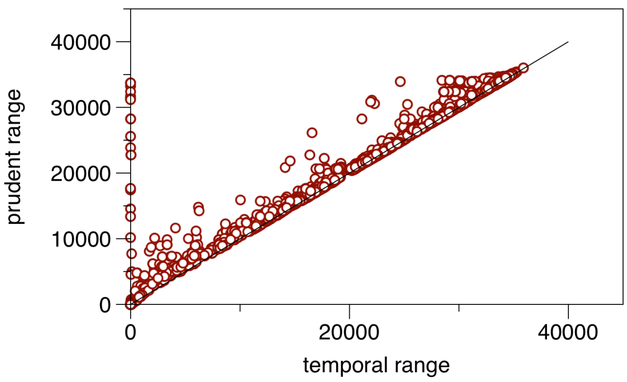

Figure F1: *Temporal range vs. prudent range for all nodes of the network. Deviation is 148 on average.*

Considering the large scale picture of the network, the long path correction does not make a significant difference. Figure F2 shows the path density of the standard approach and the path density computed using (2). The curves are almost identical.

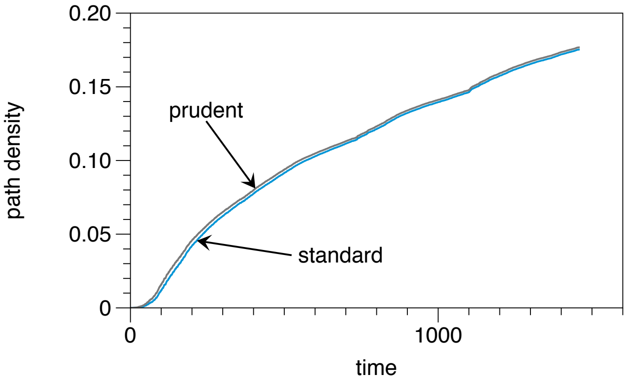

Figure F2: *Path densities for the network given by $\mathcal{A}$ (standard) and $\mathcal{B}$ (prudent).*

References

[1] P. Grindrod, M. Parsons, D. Higham, and E. Estrada. Communicability across evolving networks. *Phys. Rev. E*, 83(4):046120, Apr. 2011.

[2] R. P. Kitching. Foot and mouth disease diagnostics: requirements for demonstration of freedom from infection. In *Compendium of technical items presented to the international committee or to regional commissions*, pages 189–203, 2001.
